# Monomer unfolding of a bacterial ESCRT‐III superfamily member is coupled to oligomer disassembly

**DOI:** 10.1002/pro.5187

**Published:** 2024-10-29

**Authors:** Ndjali Quarta, Tika Ram Bhandari, Martin Girard, Nadja Hellmann, Dirk Schneider

**Affiliations:** ^1^ Department of Chemistry – Biochemistry Johannes Gutenberg University Mainz Germany; ^2^ Max Planck Institute for Polymer Research Mainz Germany; ^3^ Institute of Molecular Physiology Johannes Gutenberg University Mainz Germany

**Keywords:** ESCRT‐III, IM30, oligomerization, PspA, stability, unfolding, Vipp1

## Abstract

The inner membrane associated protein of 30 kDa (IM30), a member of the endosomal sorting complex required for transport (ESCRT‐III) superfamily, is crucially involved in the biogenesis and maintenance of thylakoid membranes in cyanobacteria and chloroplasts. In solution, IM30 assembles into various large oligomeric barrel‐ or tube‐like structures, whereas upon membrane binding it forms large, flat carpet structures. Dynamic localization of the protein in solution, to membranes and changes of the oligomeric states are crucial for its in vivo function. ESCRT‐III proteins are known to form oligomeric structures that are dynamically assembled from monomeric/smaller oligomeric proteins, and thus these smaller building blocks must be assembled sequentially in a highly orchestrated manner, a still poorly understood process. The impact of IM30 oligomerization on function remains difficult to study due to its high intrinsic tendency to homo‐oligomerize. Here, we used molecular dynamics simulations to investigate the stability of individual helices in IM30 and identified unstable regions that may provide structural flexibility. Urea‐mediated disassembly of the IM30 barrel structures was spectroscopically monitored, as well as changes in the protein's tertiary and secondary structure. The experimental data were finally compared to a three‐state model that describes oligomer disassembly and monomer unfolding. In this study, we identified a highly stable conserved structural core of ESCRT‐III proteins and discuss the advantages of having flexible intermediate structures and their putative relevance for ESCRT‐III proteins.

## 1 INTRODUCTION

The *Inner Membrane‐associated protein of 30 kDa* (IM30), also known as Vipp1 (the *vesicle‐inducing protein in plastids 1*), is found in organisms/organelles that perform oxygenic photosynthesis and contain thylakoid membranes (TMs), that is, cyanobacteria and chloroplasts (Kroll et al., 2001; Li et al., 1994; Vothknecht et al., 2012; Westphal et al., 2001). While its exact physiological function still is under debate, IM30 clearly is essential for the biogenesis and maintenance of the TMs that contain the complexes of the photosynthetic electron transfer chain (Fuhrmann, Gathmann, et al., 2009a; Junglas & Schneider, 2018; Kroll et al., 2001; Vothknecht et al., 2012; Westphal et al., 2001).

IM30 likely evolved via duplication of the gene coding for the *phage shock protein A* (PspA), a bacterial protein likely involved in maintenance and repair of the cytoplasmic membrane (Darwin, 2005; Manganelli & Gennaro, 2017; Popp et al., 2022). When compared to PspA, IM30 has a prolonged C‐terminus, which likely is a determinant for its distinct function in TM biogenesis and maintenance (Hennig et al., 2017; Vothknecht et al., 2012; Westphal et al., 2001). While in bacteria solely PspA or PspA‐like proteins, such as LiaH in *Bacillus subtilis*, are encoded and chloroplasts exclusively contain IM30, many cyanobacterial genomes encode both, PspA plus IM30 (Popp et al., 2022; Vothknecht et al., 2012).

Both PspA and IM30, together with canonical eukaryotic ESCRT‐III proteins, belong to the ESCRT‐III superfamily (Gupta et al., 2021; Junglas et al., 2021; Liu et al., 2021), where all superfamily members share key structural elements (Schlösser et al., 2023): all superfamily members have a structural core of five α‐helical segments, all contain a coiled‐coil formed by helices α1‐2, as well as have a pronounced tendency to form large oligomeric barrel/rod structures. While the sequences of IM30 and PspA of the cyanobacterium *Synechocystis* sp. PCC 6803 (from here on: *Synechocystis*) are only 51% similar (Bultema et al., 2010), the monomer structures of the proteins are highly similar (Gupta et al., 2021; Junglas et al., 2021) with a largely conserved structure of the PspA‐domain, formed by helices α0‐5. However, as mentioned before, IM30 contains an extra helix α6 at its C‐terminus.

**FIGURE 1:**
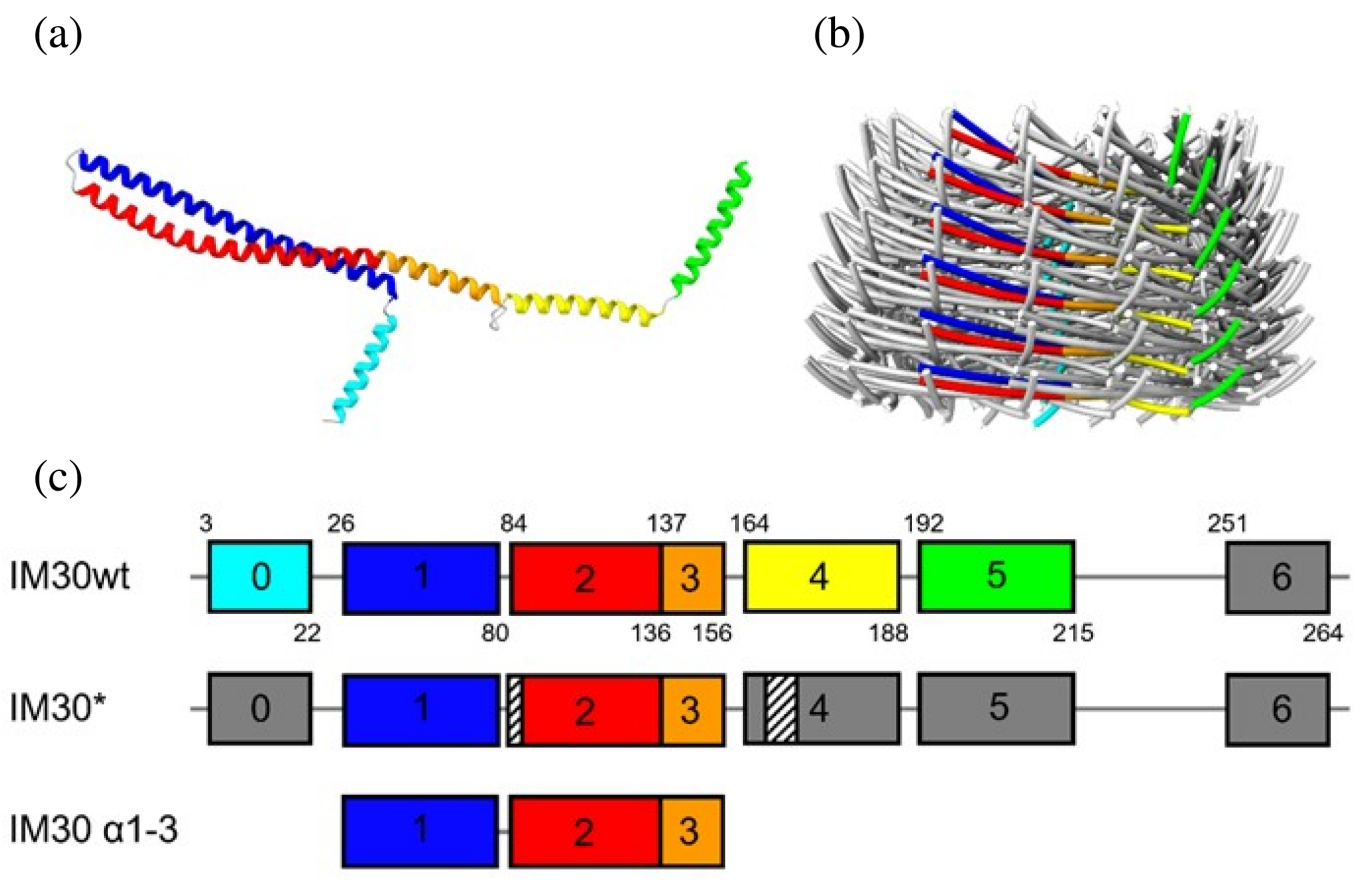
IM30 structure and schematic representations of truncated IM30 variants. (a) Structure of an IM30 monomer without the C‐terminal flexible region containing α6 (PDB: 7O3Y). The colors are as in (c). (b) Model of an IM30 barrel with the α‐helices shown as tubes, and monomers of a single stack colored as in (a) and (c) (PDB: 7O3Y). (c) Schematic representation of full‐length and truncated IM30 variants analyzed in this study. Numbers inside the boxes represent numbers of helices, and colors in boxes correspond to colors of helices in the model. Gray color indicates flexible and disordered regions. Small numbers above and below boxes represent beginning and end of regions defined in this study as helix. The position of the six mutated amino acids in IM30* (at positions 83–84 and 168–171) are indicated by white boxes with black stripes.

In solution, IM30 of the cyanobacteria *Synechocystis* and *Nostoc punctiforme* spontaneously form large homo‐oligomeric, dome‐shaped barrel structures with various symmetries, albeit the protein can also form rod structures (Fuhrmann, Bultema, et al., 2009b; Gupta et al., 2021; Hennig et al., 2015; Junglas et al., 2024; Liu et al., 2021). Within these oligomers, the PspA domains are organized in stacked layers (Figures 1a,b, S1, and S2) where the monomeric structural units of the oligomer, that is, the protomers, are connected by conserved intermolecular interfaces within one layer and between layers (Gupta et al., 2021; Junglas et al., 2021; Liu et al., 2021; Schlösser et al., 2023). Within a barrel, a single monomer can interact with up to 15 other monomers that are up to one layer away along the symmetry axis and as far as three stacks away in a different symmetrical unit (Gupta et al., 2021). The recently solved structures of two different cyanobacterial IM30 proteins confirmed that the structural features and monomer interactions within the ESCRT‐III superfamily are conserved, involving the central coiled‐coil formed by helices α1‐2, conserved flexible hinge regions, and defined inter‐and intramolecular interfaces (Gupta et al., 2021; Liu et al., 2021; Schlösser et al., 2023). When part of an oligomeric assembly, the *Synechocystis* IM30 protein is largely α‐helical, with five α‐helices (helices α0‐5) being resolved in the cryo‐EM structures (Gupta et al., 2021). Yet, in an IM30 variant that does not form stable homo‐oligomeric structures anymore (IM30*), the regions forming helices α0 plus α4‐7 in the oligomer appear to be unstructured, whereas the coiled‐coil formed by helices α1‐2, as well as helix α3, which is a prolongation of α2, remain structured (Junglas et al., 2020). This observation indicates that ring disassembly coincides with partial unfolding of IM30 monomers, or, vice versa, ring formation involves the formation of α‐helical segments in previously disordered regions. However, IM30* contains mutations that disturb inter‐monomer contacts, and it can currently not be excluded that these mutations might also destabilize the protein secondary structure (Junglas et al., 2020).

Yet, members of the ESCRT‐III superfamily do not only share common structural features (Schlösser et al., 2023), all members of the ESCRT‐III superfamily appear to be involved in membrane remodeling processes (Carlton & Baum, 2023; Heidrich et al., 2017; McCullough et al., 2018; Siebenaller & Schneider, 2023; Zhen et al., 2021). IM30 binds to negatively charged membrane surfaces (Heidrich et al., 2016; Hennig et al., 2015; Theis et al., 2019; Thurotte & Schneider, 2019) and forms membrane‐covering carpets (Junglas et al., 2020, 2024) or spiral‐like structures (Naskar et al., 2023; Pan et al., 2024) in vitro. The formation of carpets has been shown to involve (i) adhesion of IM30 barrels at membrane surfaces and (ii) subsequent disassembly of the barrels (Junglas et al., 2020). How exactly these observations relate to the in vivo function of IM30 is still largely unknown, although membrane binding as well as formation of large membrane‐attached IM30 assemblies has been observed in vitro upon membrane stress (Bryan et al., 2014; Gutu et al., 2018; Zhang et al., 2016). For ATP production driven by the pH gradient established across the TM in the light, the integrity of the TM must be maintained, and IM30 carpets were suggested to protect membranes against proton leakage (Junglas et al., 2020; Siebenaller et al., 2020). Interestingly, IM30* forms carpets rapidly and appears to protect membranes more effectively than IM30 wt, at least in vitro (Junglas et al., 2020), indicating that IM30 oligomerization counteracts membrane binding and protection (Heidrich et al., 2016). Thus, the availability of mono‐ vs. oligomeric IM30 likely needs to be fine‐tuned in vivo. The structural dynamics (monomer vs. oligomer) as well as soluble vs. membrane‐attached IM30 populations are likely crucial for the IM30 in vivo function (Siebenaller et al., 2019). In fact, soluble as well as membrane‐attached IM30 fractions have been observed in vivo (Bryan et al., 2014; Gutu et al., 2018).

Very little is known about the processes controlling IM30 oligomer formation. Since the IM30 function appears to be coupled to its dynamic cellular localization, involving different IM30 folding states (Junglas et al., 2020; Siebenaller et al., 2019), it is crucial to understand the structural dynamics in greater detail to be able to eventually control the formation of specific IM30 states for detailed in vitro analyzes.

Thus far, the impact of the IM30 oligomeric state on its in vitro and in vivo activity has been studied mainly via generation and analyzes of truncated IM30 variants as well as variants carrying destabilizing mutations (Hennig et al., 2017; Junglas et al., 2020; Thurotte & Schneider, 2019). Yet, to better understand the thermodynamic principles guiding oligomer formation in solution and/or on membrane surfaces, it clearly is advantageous to understand oligomerization of the wt protein. Luckily, after unfolding IM30 by urea, the protein refolds, forms the prototypical IM30 oligomeric structures and binds to lipid membranes as observed with the protein purified under native conditions (Heidrich et al., 2018; Hennig et al., 2017; Junglas et al., 2024; Siebenaller et al., 2021). These observations now enabled us to study individual steps during urea‐mediated unfolding of IM30 (oligomers).

We now show that unfolding of a significant, mostly α‐helical part of wt IM30 monomers is coupled to disassembly of the large, homo‐oligomeric ring structures. Yet, when the oligomeric IM30 barrel structures disassemble into smaller oligomers and/or monomers, the structure of the α1‐2 coiled‐coil is retained. Based on our experimental data and a derived theoretical model we demonstrate that it is impossible to concurrently disassemble all oligomeric barrel structures completely without simultaneously unfolding a fraction of the IM30 monomers. Eukaryotic ESCRT‐III proteins are also known to form oligomeric structures that are dynamically assembled from monomeric/smaller oligomeric proteins, a process that is still poorly understood. Based on our analysis of a manageable ESCRT‐III model, we here discuss the advantages of having a flexible intermediate structure and its potential relevance for ESCRT‐III superfamily proteins.

## 2 RESULTS AND DISCUSSION

### 2.1 Coarse‐grained, in silico unfolding of IM30

Based on previous observations, it is feasible to assume that the structure of IM30* mimics the structure of the IM30 wt protein when the protein is not part of an oligomeric assembly. Yet, a direct comparison with the structure of the IM30 wt in a monomeric form is not possible, since this cannot be isolated under native conditions due to its strong intrinsic propensity to homo‐oligomerize (Figure 1).

**FIGURE 2:**
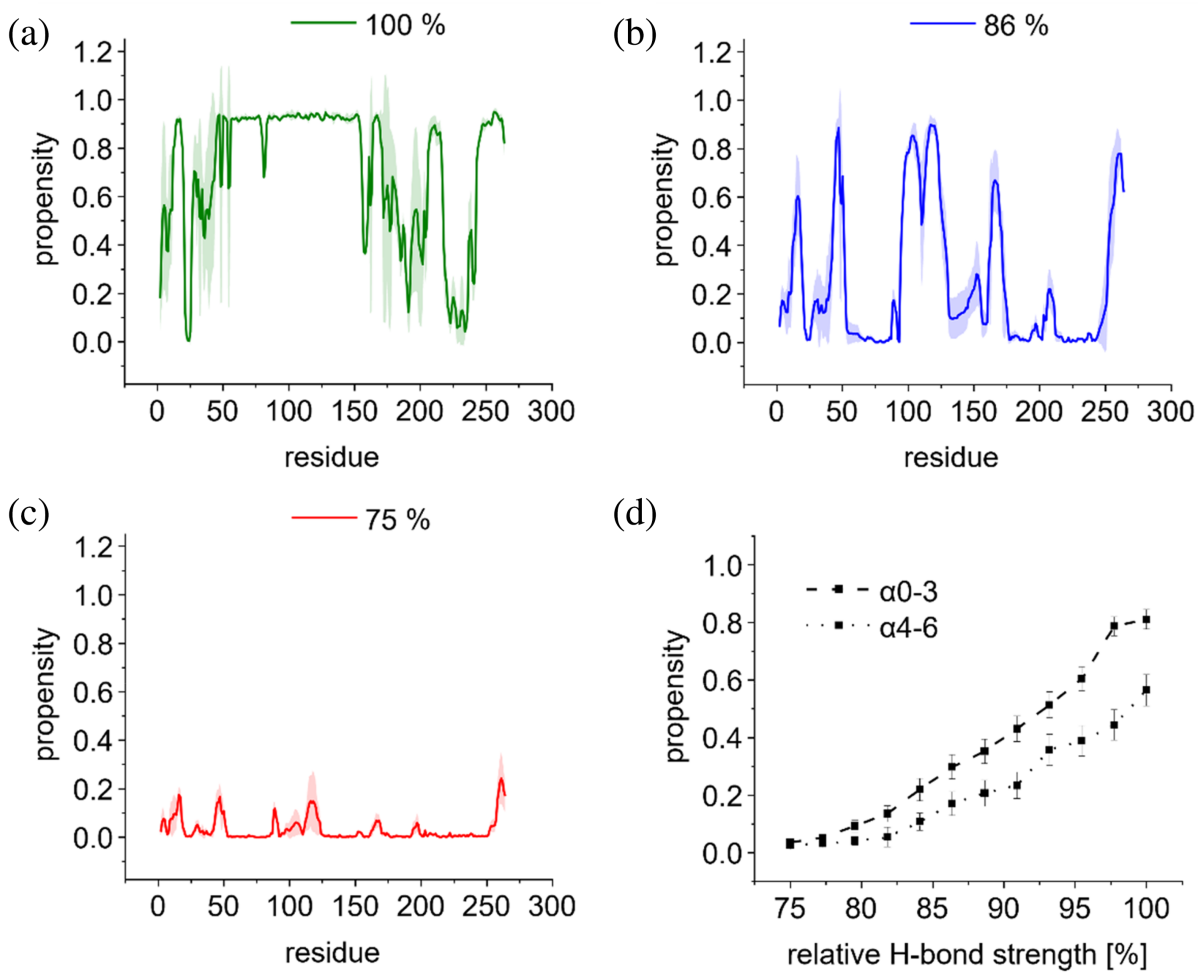
Influence of relative H‐bond strength on the α‐helicity of IM30 monomers determined via coarse‐grained simulations in solution. Helical propensity versus amino acid position of full‐length protein, at (a) 100%, (b) 86%, and (c) 75% H‐bond strength. (d) Helical propensity of α0‐3 and α4‐6 within the full length α0‐6 monomer structure.

Therefore, we first utilized the power of molecular dynamics (MD) simulations to precisely follow the unfolding transition of the mainly α‐helical IM30 wt structure upon in silico destabilization. Since H‐bonds are the main interactions stabilizing α‐helices, we performed MD simulations at decreasing H‐bond strength to destabilize the protein secondary structure and followed the resulting changes in the propensity of individual amino acids to be part of an α‐helix. Numerically, this was done by reducing the H‐bond potential (see Section 4.7). At 100% H‐bond strength, 60% and 80% of the α1‐3 and α4‐6 regions, respectively, were α‐helical, and by lowering the H‐bond strength to 75%, the α‐helical propensity was drastically reduced to 10% (Figure 2).

In addition, our analyses suggested that the coiled‐coil‐forming region α1‐3 has a consistently higher propensity to form α‐helices compared to α4‐6 at equal H‐bond strength, indicating a higher stability of the helices involved in coiled‐coil formation (Figure 2d).

Furthermore, at equal H‐bond bias the helical propensities were only slightly reduced for the IM30* variant when compared to their counterparts in IM30 (Figure S3), which supports the assumption that IM30* properly represents the non‐oligomerized, monomeric IM30 wt protein. These observations prompted us to experimentally investigate the changes in the IM30 structure upon disassembly of higher‐ordered IM30 oligomers. We assumed that our MD results can largely be linked to experimental data, when in vitro protein unfolding by urea mainly proceeds via destabilization of H‐bonds (Lim et al., 2009), and not the solvent quality.

### 2.2 Urea‐induced destabilization of the IM30 oligomer structure

Due to our simulation results and the low helix‐forming propensity of the C‐terminal helices α4‐6 (Junglas et al., 2020; Thurotte & Schneider, 2019), structure formation of this part of the protein might be coupled to homo‐oligomerization of the full‐length protein, that is, to the formation of stabilizing contacts. Thus, to determine whether (partial) unfolding of the IM30 wt monomer precedes, is paired with, or follows oligomer disassembly, we monitored changes in the secondary and tertiary structure as well as in the oligomeric assembly state at urea concentrations in the range 0–7M.

**FIGURE 3:**
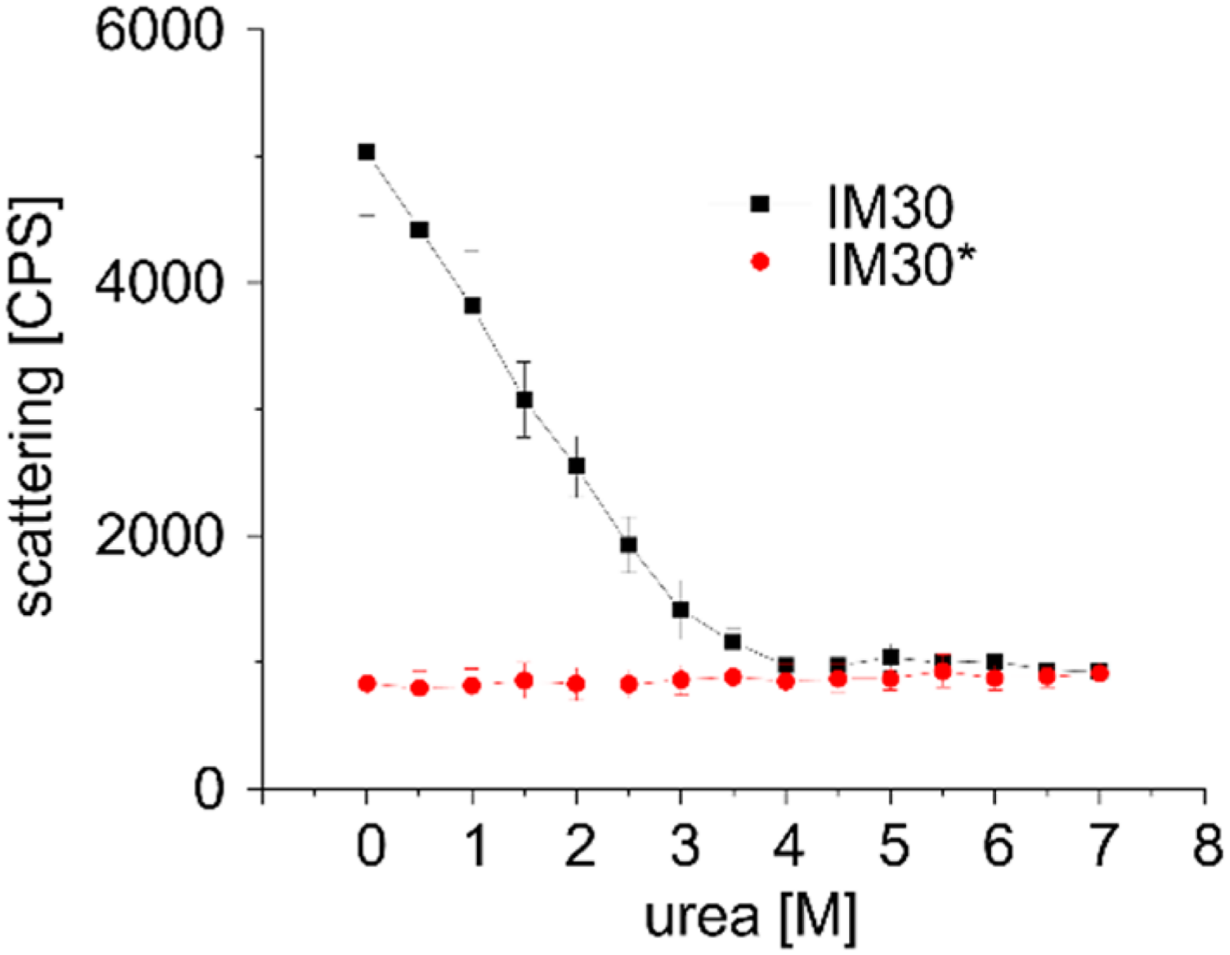
Scattering signal of IM30 at increasing urea concentrations. IM30 wt (black squares) and IM30* (red circles). The *y*‐axis shows counts per second (CPS). The error bars represent SD, *n* = 3.

To first monitor changes in quaternary structure, we measured the scattering signal of IM30 wt at increasing urea concentrations. Due to the large size, IM30 barrels show a strong scattering signal in the absence of urea (Figure 3).

Upon increasing the urea concentration from 0 to 3.5M urea, the scattering signal decreased nearly linearly until it reached a constant level which was about six times lower than the scattering signal observed at 0M urea. This suggests that the barrel structure is continuously destabilized until a concentration of ~3.5M urea is reached. For a well‐defined system with only one type of oligomer, a sigmoidal curve for disappearance of the oligomer in presence of a chemical denaturant might be observed (as e.g., described for oligomeric hemocyanin (Hübler et al., 1998)). However, IM30 forms barrels of different sizes, as well as stacks of barrels and even tubular structures (Fuhrmann, Bultema, et al., 2009b; Junglas et al., 2024; Saur et al., 2017). Therefore, multiple equilibria between various oligomeric states exist, leading to a series of transitions which cannot be individually resolved anymore. In contrast to the wt, the scattering signal determined for the IM30* variant that does not form any large oligomers, remains essentially constant at all urea concentrations and is equal to the level that the wt protein reached at urea concentrations >3.5 M.

### 2.3 Monitoring IM30 denaturation via changes in Trp fluorescence emission

Changes in Trp fluorescence characteristics can be used to monitor variations in the polarity of a Trp's environment, such as exposure of Trp residues to a more polar aqueous environment due to protein unfolding, leading to a redshift of Trp's emission maximum (Hellmann & Schneider, 2019). Therefore, we measured Trp fluorescence emission spectra at increasing urea concentrations to monitor alterations of the Trp environment, induced by changes in the tertiary and/or quaternary structure of IM30.

**FIGURE 4:**
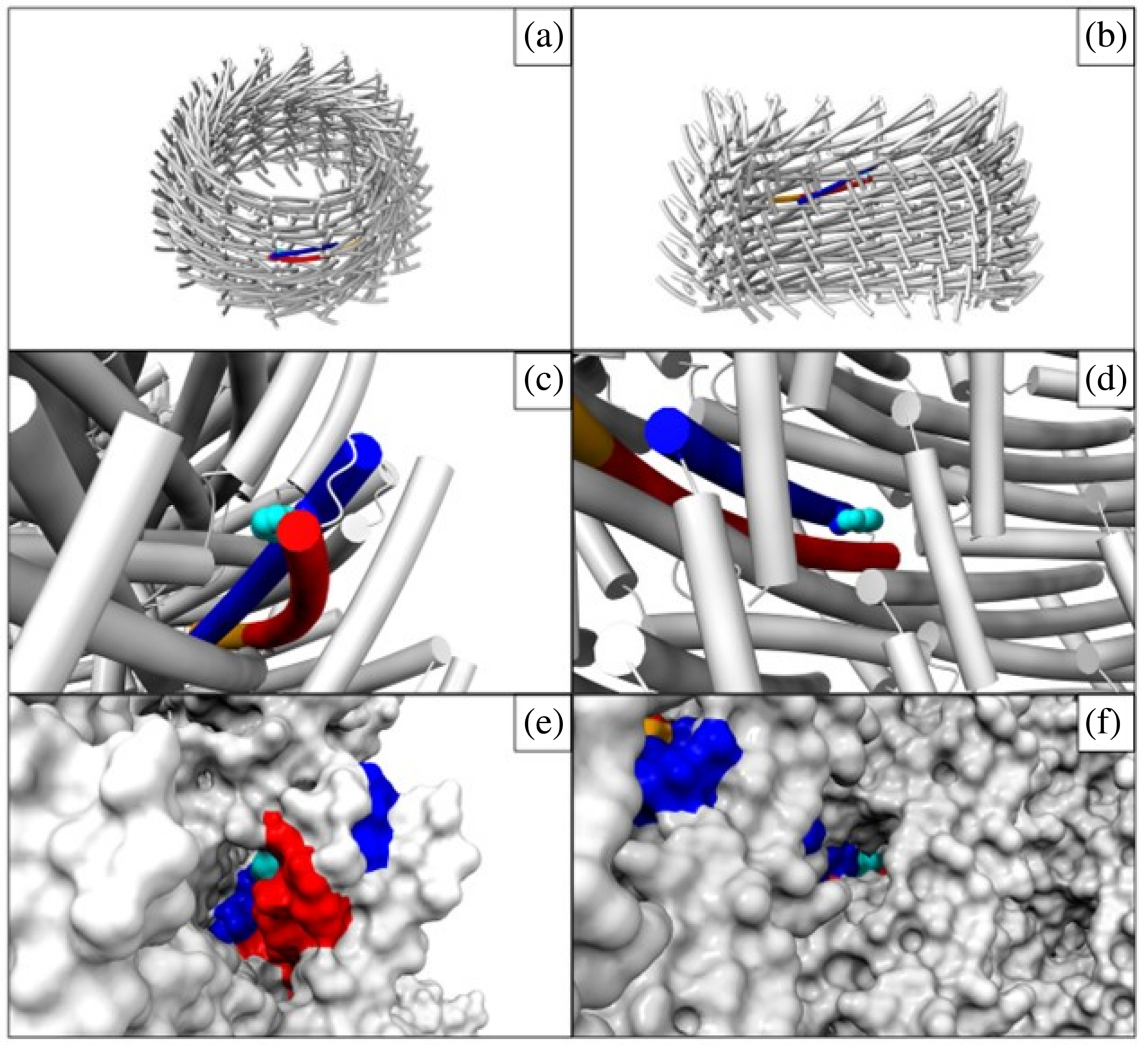
The IM30 Trp71 pocket. (a) Location of a coiled‐coil in layer 3 of the IM30 barrel structure in tube representation (PDB: 7O3Y). (b) Model of IM30 a barrel in tube representation in the same orientation as in Figure 1b, yet with seven vertical monomer stacks being removed to yield a view into the inside of the barrel structure. See Figures S1 and S2 for details on the arrangement of monomers in the barrel as stacks and layers. (c–f) Solvent access to the Trp71 pocket as seen from the outside (c + e) and inside (d + f) in tube representation (c + d) and surface representation (e + f). α‐Helices α1 (blue), α2 (red), and α3 (orange). Trp side chain atoms are shown as spheres, and the indole ring atoms and its corresponding surface are colored in cyan.

IM30 contains a single Trp residue in position 71 on helix α1 (Figure 4). This residue is part of the structured core of IM30, that is, the coiled‐coil formed by helices α1 and α2, and is facing away from the barrels outer surface to the inside of the barrel.

In the coiled‐coil, Trp71 is sandwiched between the helices α1 and α2. In the barrel, the accessibility of Trp71 is further confined by protomers of neighboring layers. Residues from these neighboring protomers form together a small pocket around Trp71, from here on referred to as the Trp‐pocket. Thus, in the IM30 wt barrel structures Trp71 is primarily confined by the formation of the coiled‐coil plus further buried in a Trp‐pocket formed by multiple protomers (Figure 4).

Although the Trp residue is somewhat buried within the barrel, solvent can access the Trp pocket from both the outside and the inside of the barrel (Figure 4e,f). From the outside, solvent access is provided in all layers through a large gap near the closed end of the coiled‐coil hairpin below the adjacent α5 of the monomer *i* ^+3^ (Figure 4c,e), another gap between α2 of the coiled‐coil and α3 of monomer *i* in layer L^−1^ and also between α3 and α2 of monomer *i* in layer L^+1^. From the inside there is solvent access to the Trp pocket through an elongated opening (Figure 4d,f) that is also found in all layers of the barrel structure.

When calculating the solvent‐accessible surface area (SASA) of the Trp's indole ring in the barrel structure (78 Å^2^), the isolated coiled‐coil (100 Å^2^) and α2 by itself (166 Å^2^), it becomes evident that the SASA of the Trp increases only by 28% upon barrel disassembly, when the coiled‐coil is still intact (78 Å^2^ → 100 Å^2^), compared to an increase of >100% in the isolated α2 helix (78 Å^2^ → 166 Å^2^). It has been shown by MD simulation that the wavelength of maximal Trp emission correlates with the solvent accessible area (Lopez & Martínez, 2018). Therefore, we expect that barrel disassembly has a much smaller effect on Trp's emission spectrum than opening and/or unfolding of the coiled‐coil.

**FIGURE 5:**
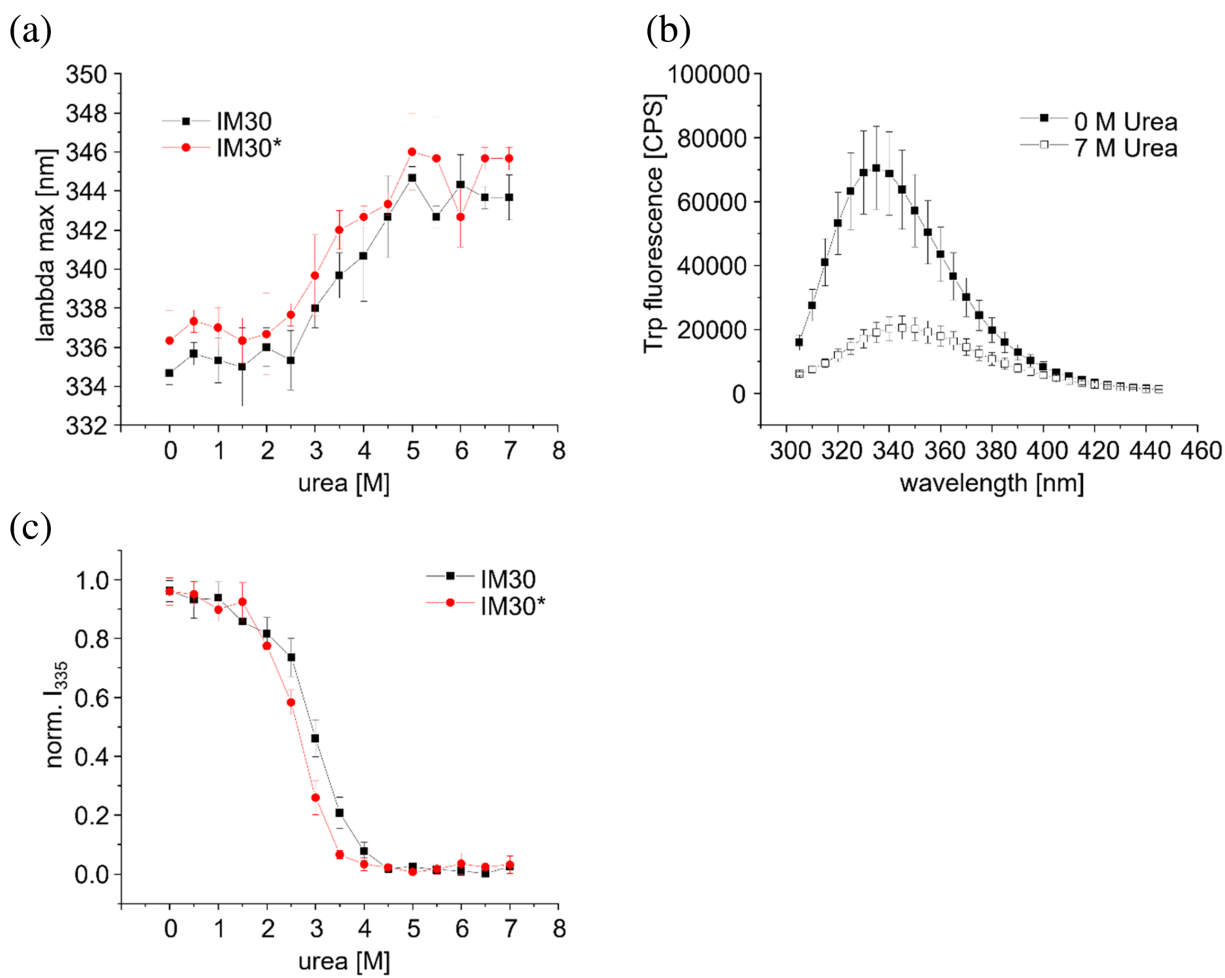
Trp fluorescence emission of IM30 at increasing urea concentrations. (a) Wavelength of the IM30wt (black squares) and IM30* (red circles) fluorescence emission maximum at increasing urea concentrations. (b) Trp fluorescence emission spectra of IM30 at 0 M urea (full squares) and 7 M urea (empty squares). (c) Normalized fluorescence intensity at 335 nm observed for IM30wt (black squares) and IM30* (red circles) at increasing urea concentrations. See Figure S4 for non‐normalized data. The error bars represent SD, *n* = 3.

This assumption is supported by the observation that the fluorescence emission maximum of IM30 wt is only slightly lower than that of IM30* (334.7 ± 0.6 nm and 336.3 ± 1.5 nm, respectively), while the emission maximum of the unfolded protein is strongly increased (344 ± 1 nm) (Figure 5a). Also, the fluorescence intensity is strongly decreased at a urea concentration of 7M compared to the intensity in absence of urea (Figure 5b,c).

In order to monitor urea‐induced denaturation of IM30 wt and IM30*, the relative intensity at 335 nm (*I*_335_) and the position of the emission maximum (*λ*_max_) were monitored (Figure 5a,c). The decrease in the Trp fluorescence intensity and the shift in *λ*_max_ (Figure 5a,c) can be divided into three stages for both variants: at low urea concentrations from 0 to 2M, the Trp fluorescence intensity decreases moderately. The relative intensity is about 85 ± 5% for IM30 and 80.6% ± 0.6% for IM30*. From 2.5 to 4.5M urea a pronounced change is observed, whereas above 4.5M urea the characteristics of the Trp's environment appear not to change further. The changes of *λ*_max_ at increasing urea concentrations follow a similar pattern, yet between 0 and 2M urea the *λ*_max_ value does not change at all. The pronounced change in Trp's spectral characteristics between 2.5 and 4.5M urea indicates a considerable exposure of Trp71 to a more polar environment, likely due to unwinding of the helix α1/2 coiled‐coil (as further analyzed and discussed below).

Taken together, these observations indicate that the urea‐induced solvent exposure of Trp71 is similar for IM30 wt and IM30*, with a slight right‐shift of the curve in case of IM30 wt, which might be due to the stabilization of the protein structure by barrel formation.

### 2.4 Urea‐induced destabilization of the IM30 secondary structure

To next analyze whether between 2.5 and 4.5M urea mainly the coiled‐coil helices unfold, as hypothesized based on the fluorescence measurements, CD‐spectroscopy was employed, allowing to monitor changes in the protein's secondary structure.

**FIGURE 6:**
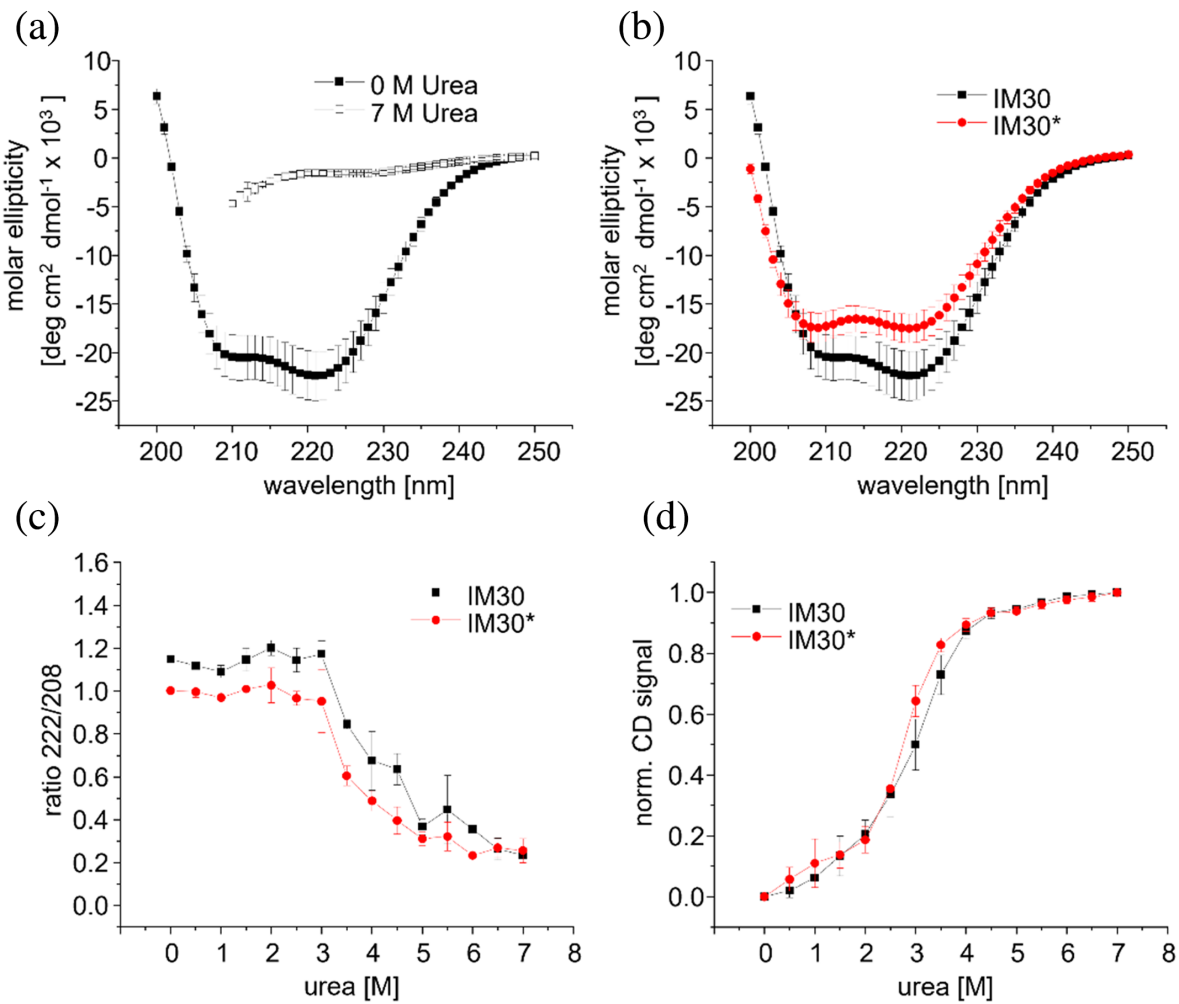
Alterations in the CD signal at increasing urea concentrations. (a) CD spectra of IM30 wt at 0M urea (full squares) and 7M urea (empty squares). (b) CD spectra of IM30 wt (black squares) and IM30* (red circles) at 0M urea; (c) ratio of the CD signal at 222–208 nm at increasing urea concentrations for IM30 wt (black squares) and IM30* (red circles). (d) Normalized CD signal at 222 nm at increasing urea concentration for IM30 wt (black squares) and IM30* (red circles). See Figure S4 for non‐normalized data. The error bars represent SD, *n* = 3.

In the absence of urea, the IM30 wt protein shows a strong CD‐signal with minima at 208 and 222 nm, as characteristic for a mainly α‐helical protein (Figure 6a), in line with the solved structure (Gupta et al., 2021). The shape of the spectrum is different for IM30* when compared to IM30 wt (Figure 6b), and the fraction of protein adopting an α‐helical structure is smaller in IM30*, in agreement with earlier studies (Junglas et al., 2020). The difference in shape is also reflected in the different value for the ratio of the CD signals at 222 and 208 nm in absence of urea (Figure 6c), which illustrates changes in the general shape of the CD spectrum. The overall dependence of the CD signal at 222 nm on the urea concentration is quite similar to the changes of the Trp fluorescence emission observed at increasing urea concentrations (Figure 6d), both for IM30 wt and IM30*: between 0 and 2M only a small change is observed, followed by a pronounced decrease in ellipticity between 2.5 and 4.5M, and a constant ellipticity above 4.5M urea.

For both proteins, the ratio of the CD‐signals at 208 and 222 nm remains essentially constant between 0 and 2M urea (Figure 6c) and starts to drop at about 2.5M urea. Of note: the corresponding ratio determined at urea concentrations >4.5M were excluded from the analysis and discussion, due to a low signal‐to‐noise ratio at 208 nm caused by high absorbance of urea as indicated by high detector voltages.

Together, these observations indicate that for both IM30 wt and IM30* between 2.5 and 4.5M urea most α‐helical regions unfold. As in IM30* helices α0 and α4‐6 are largely disordered (Junglas et al., 2020), the unfolding curve likely is dominated by the coiled‐coil formed by helices α1‐3.

### 2.5 Coarse‐grained, in silico unfolding of isolated IM30 regions

To test our assumption that the coiled‐coil forms a stable structured core whereas the C‐terminal region is disordered upon barrel disassembly, we next carried out coarse grained simulations of isolated protein segments while varying the H‐bond strength in our simulation, mimicking the destabilizing effect of urea.

**FIGURE 7:**
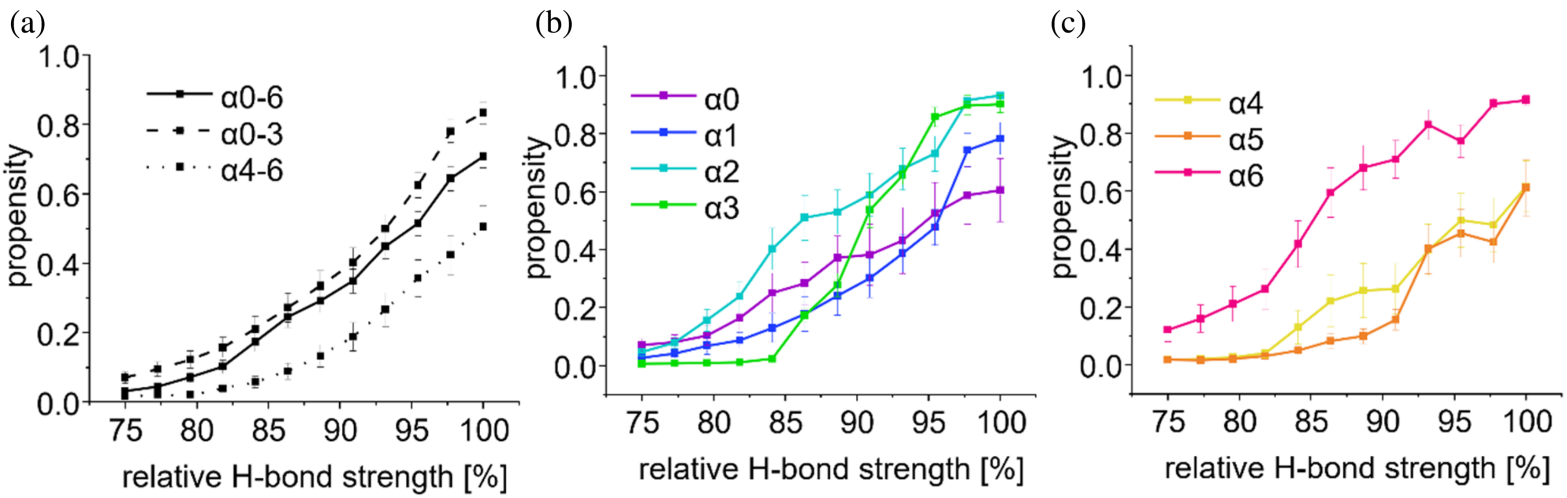
Influence of relative H‐bond strength on the α‐helicity of IM30 determined via coarse‐grained simulations of monomers in solution. (a) Helical propensities of the full length (α0‐6) IM30 wt and fragments α0‐3 and α4‐6 simulated separately at varying H‐bond strengths. (b) Helical propensity of each helix of the α0‐3 fragment with respect to relative H‐bond strength calculated based on simulations of the α0‐6 full‐length structure. (c) Helical propensity of each helix within the α4‐6 fragment with respect to relative H‐bond strength calculated based on simulations of the α0‐6 full‐length structure.

Again, with decreasing relative H‐bond strengths we observed a general decrease in the propensity to form α‐helices and the decrease consistently required a stronger reduction in H‐bond strength for the α0‐3 region compared to α4‐6 (Figure 7a). This effect was observed in simulations of both the full‐length protein as well as the individual truncated variants (α0‐3 and α4‐6) (Figures 2d and 7a). Essentially identical trends were observed when the IM30* variant was analyzed, with a slightly reduced helical propensity compared to the wt IM30 (Figure S5a).

Next, we analyzed the stability of individual regions that form α‐helices in the barrel structure at varying H‐bond strength. The helices of the individually simulated N‐terminal regions show an α‐helical propensity above 40% at 92% H‐bond strength (Figure 7b), in contrast to the individually simulated C‐terminal helices α4‐6 where the α‐helicity is already drastically decreased at 92% H‐bond strength compared to 100% (Figure 7c). Essentially identical trends are observed for IM30* (Figure S5b,c). Taken together, our simulations support the assumption that IM30 retains its coiled‐coil structure while other regions of the protein become disordered upon barrel disassembly.

### 2.6 The IM30 coiled‐coil core remains intact upon barrel disassembly

The above presented results and simulations indicate that at low urea concentrations for both, IM30 and IM30*, a state with a structured α‐helical region exists. The central coiled‐coil formed by helices α1‐3 is a conserved structural motif found in all members of the ESCRT‐III protein superfamily. This coiled‐coil is the only region that remains structured in IM30*, where most of the protein is disordered in solution (Junglas et al., 2020).

In order to test the assumption that between 2.5 and 4.5M urea unwinding and unfolding of this structured core is observed independently of helices α0 and α4‐α6, we next analyzed the stability of the isolated helical hairpin formed by the helices α1‐3 (IM30_26–156_), to investigate its contributions to the structure and stability of the full‐length IM30 monomer.

**FIGURE 8:**
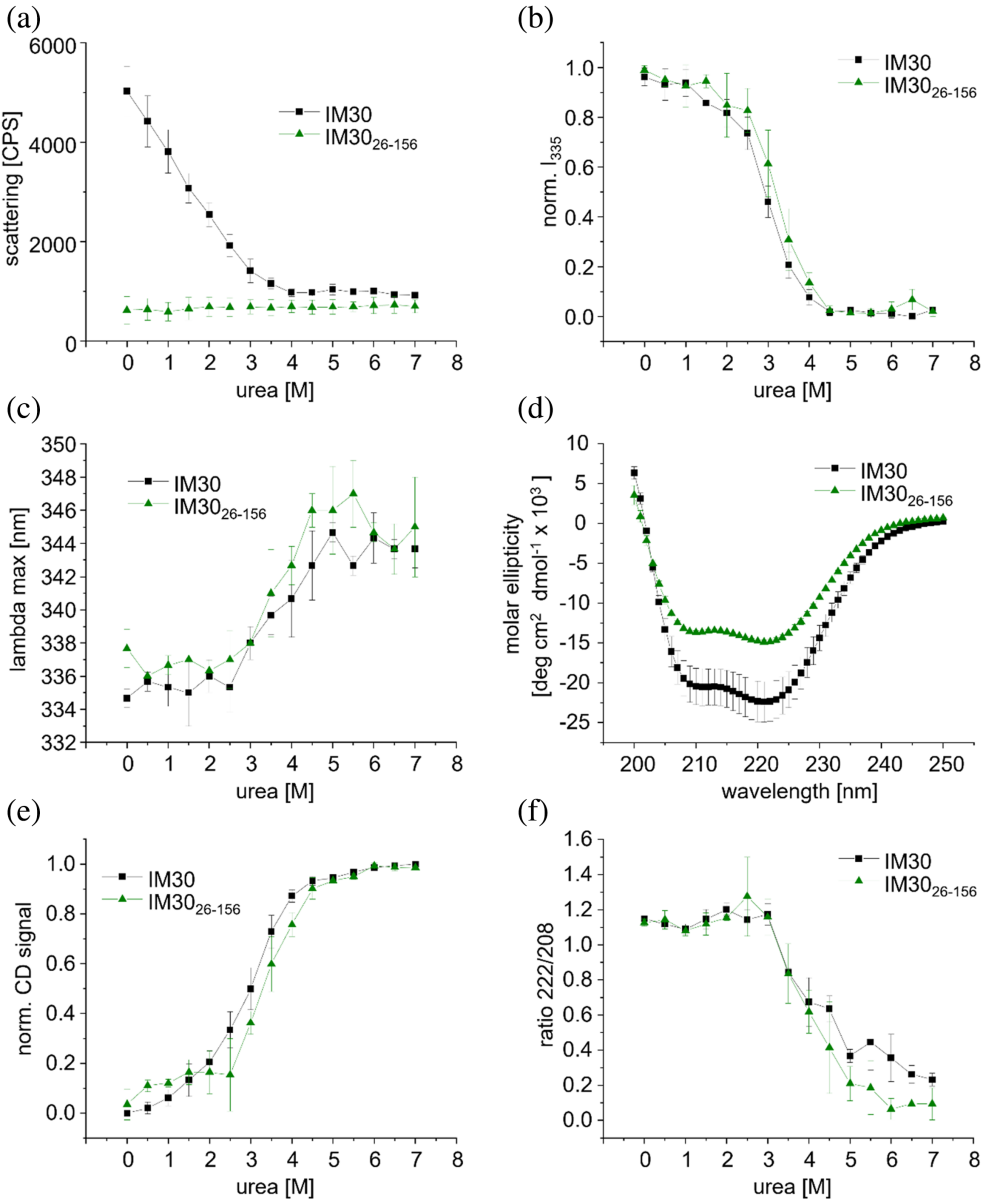
The structure of IM30_26–156_ at increasing urea concentrations. (a) Scattering, (b) normalized Trp Fluorescence intensity at 335 nm, (c) maximum wavelength, (d) CD spectrum, (e) normalized CD signal at 222 nm, and (f) CD signal ratio 222 ‐to‐208 nm at increasing urea concentrations for IM30 (black squares) and IM30_26–156_ (green triangles). See Figure S4 for non‐normalized data. The error bars represent SD, *n* = 3.

The scattering signal of IM30_26–156_ was as low as observed before for IM30* (Figure 3) and remains constant in the range of 0–7M urea (Figure 8a). This is perfectly in line with the simulations (Figure 7) and the recent observation that the isolated coiled‐coil does not form large oligomeric structures in solution (Junglas et al., 2020; Thurotte & Schneider, 2019). The Trp fluorescence emission characteristics of IM30_26–156_ changed only slightly at low urea concentrations followed by a strong decrease in intensity and a red‐shift in the fluorescence emission maximum at concentrations above 2.5M urea (Figure 8b,c), exactly as observed before for the wt and IM30* variant (Figure 5). This strongly supports the conclusion that the Trp71 environment inside the isolated coiled‐coil remains largely unaltered at low urea concentrations (0–2.5M), whereas the Trp environment becomes more polar at higher urea concentrations due to exposure of Trp71 to the aqueous solution caused by unwinding and possible unfolding of the coiled‐coil, visible as a red shift in the Trp emission maximum. After a strong change in the fluorescence characteristics between 2.5 and 4.5M urea, the fluorescence intensity and the fluorescence emission maximum remained constant from about 4.5M urea on, as also observed for IM30 and IM30* (Figure 5). This indicates that increasing the urea concentration did not result in further protein unfolding. Unfolding of the coiled‐coiled structure between 2.5 and 4.5M urea was also shown by CD‐spectroscopy, indicated by a very sharp decrease in the amplitude of the CD‐signal at 222 nm (Figure 8e). Again, as observed for the other two variants, the decrease in the CD‐signal amplitude at 222 nm is mirrored by the decrease in the Trp emission intensity, strongly supporting the hypothesis that the loss of tertiary interactions in the coiled‐coil coincides with the loss of remaining α‐helical structure in IM30. In fact, also the CD‐signal ratio of 222‐to‐208 nm decreased (Figure 8f), as observed for IM30 and IM30* at similar concentrations (Figure 6d). A 222/208 ratio >1 is a well‐known indication for coiled‐coil formation, or, more generally, for interaction of α‐helices (Holtzer & Holtzer, 1995; Monera et al., 1993), and thus, the coiled‐coil appears to unwind at urea concentrations >3M (Figure 8f). Unwinding of the coiled‐coil, which leads to solvent exposure of Trp71, is coupled to unfolding of the α‐helices (Figures 5, 6, and 8). As for IM30 and IM30*, the CD‐signal at 222 nm reached a plateau at ~4.5M urea where the coiled‐coil‐forming helices are entirely unfolded.

Together, the analyses of the isolated helical hairpin strongly support the assumption that the helix α1‐3 coiled‐coil is the most stable structure in the wt protein and only unfolds at high urea concentration, that is, at conditions where IM30 oligomers are already disassembled and helices α0 and α4‐α6 are unfolded.

### 2.7 Barrel disassembly of IM30 wt is rate limiting in unfolding kinetics

While the scattering data indicate that disassembly of IM30 wt barrels is nearly completed at urea concentration <3M, the fluorescence and CD data suggest unfolding of α‐helices at urea concentrations >3M, indicating that barrel disassembly must take place before the tertiary structure around Trp71 changes.

**FIGURE 9:**
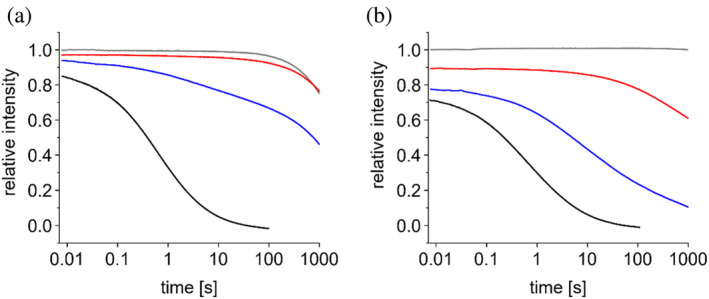
IM30 wt denaturation kinetics. IM30 wt was mixed 1:1 with a solution containing different urea concentrations, resulting in the following final concentrations: gray: 0M (control); red: 2M; blue: 3M; black: 4M urea. The signal of the control at the beginning of the reaction was set to 1, the signal at 100 s measured in presence of 4M urea was defined as end of the reaction and set to zero. (a) Changes in the Trp‐fluorescence; (b) changes in the scattering intensity. The decrease in the Trp‐fluorescence intensity in the control starting at around 100 s is caused by photobleaching. One typical result is shown. Individual curves are the mean of 2–3 repetitions. The results of experiments with three different protein preparations are shown in Figure S6.

To further scrutinize this assumption, we next investigated the kinetics of unfolding IM30 or IM30*, respectively. To this end, solutions containing the protein in buffer were rapidly mixed 1:1 with a solution containing 8, 6, or 4M urea. The reaction was monitored on one hand based on Trp fluorescence as indicator for tertiary structure changes and on the other hand by measuring the change in scattering to follow barrel disassembly (Figure 9). To determine the respective signal of the initial, native state, the proteins were mixed with urea‐free buffer in a separate experiment (control).

As can be seen in Figure 9, the Trp fluorescence emission and the scattering intensities quickly decrease in presence of 4M urea. The denaturation kinetics of IM30 wt clearly slows down with decreasing urea concentrations. In case of the Trp fluorescence intensity, photo‐bleaching is observed at longer times as a decrease in intensity of the control (0M urea), and the small change in the Trp fluorescence observed in presence of 2 M urea is barely distinguishable from the control, that is, neglectable. In contrast, the scattering signal is clearly distinguishable from the control. Overall, it seems that the scattering signal decreases faster than the fluorescence signal after mixing IM30 with 2 or 3M urea. As our previous results indicated that under equilibrium conditions a IM30*‐like state accumulates at intermediate urea concentrations, it might be possible that a slow denaturation of this species causes the lag observed at 2M urea. However, since denaturation of IM30* is very fast at all urea concentrations investigated, as indicated by the rapid decrease in Trp‐fluorescence within the deadtime of the instrument (Figure S7), this process cannot be responsible for the observed lag. Thus, when considering at least one oligomeric species, a folded IM30*‐like monomer, plus an unfolded monomer, the dissociation of the oligomer appears to be the rate limiting step. However, there seems to be an oligomer population which dissociates very fast, as indicated by the immediate drop in scattering. The starting solution contains rings of different sizes, and also stacks of these rings (Gupta et al., 2021; Heidrich et al., 2018; Junglas et al., 2024; Saur et al., 2017; Siebenaller et al., 2019; Thurotte & Schneider, 2019; Westphal et al., 2001). In fact, the very fast initial drop in scattering likely results from stack‐disassembly. If the subsequent decrease of the scattering signal reflects dissociation of the actual ring, the Trp‐fluorescence should strictly follow the scattering signal, due to fast dissociation of the IM30*‐like intermediate (see above). Since for example, at 3M urea, most of the oligomers vanished at equilibrium, but still 40% of the fluorescence signal was maintained, the final level (relative to the maximal change possible) will be different for the fluorescence and the scattering signal. Thus, to compare the shape of the curves, the data were normalized to 1 at 6 ms and 0 at 100 s (Figure S8). We choose 100 s to keep the contribution of the bleaching signal low, which otherwise would artificially alter the shape of the Trp fluorescence curves. The resulting curves do not completely overlay, yet no consistent pattern is observable. Thus, there is no clear indication for the presence of a monomer (or small oligomeric) state with higher stability than an IM30*‐like intermediate. However, we can also not exclude that such a state exists, for example, at 2M, since the total signal change in the Trp‐fluorescence signal is very small here (even under equilibrium conditions, the fluorescence decreased only by <20%).

### 2.8 A 3‐state model describes IM30 unfolding

The experimental data indicate that denaturation of IM30 wt occurs in two major steps: (i) oligomer disassembly at low urea concentration and (ii) monomer unfolding at higher urea concentrations. To support this interpretation, we analyzed our experimental data based on a two‐step model. In this model, the different species adopted by the wt protein are the native, oligomeric state (N), an intermediate state (I) and a completely unfolded state (U). Thus, in our model IM30 wt exists in three distinct states and transitions from its native state (N) to the unfolded state (U) in two consecutive steps with the state (I) as an intermediate.

In the analyses, we assume that IM30 is essentially exclusively present in higher oligomeric forms and used the measured relative scattering signals as approximation for the fraction of barrel still present (N). With this approach, barrel disassembly itself does not have to be modeled and is instead included as the decreasing fraction of protein in state (N), which is set equal to the normalized scattering signal.

The ratio between the intermediate state (I) and the unfolded state (U) is defined by an equilibrium constant *K*, which depends on the urea concentration (see Section 4.6). The urea concentration at which *K* = 1, thus I and U are present at equal concentrations, is referred to as *c*_50_.

**FIGURE 10:**
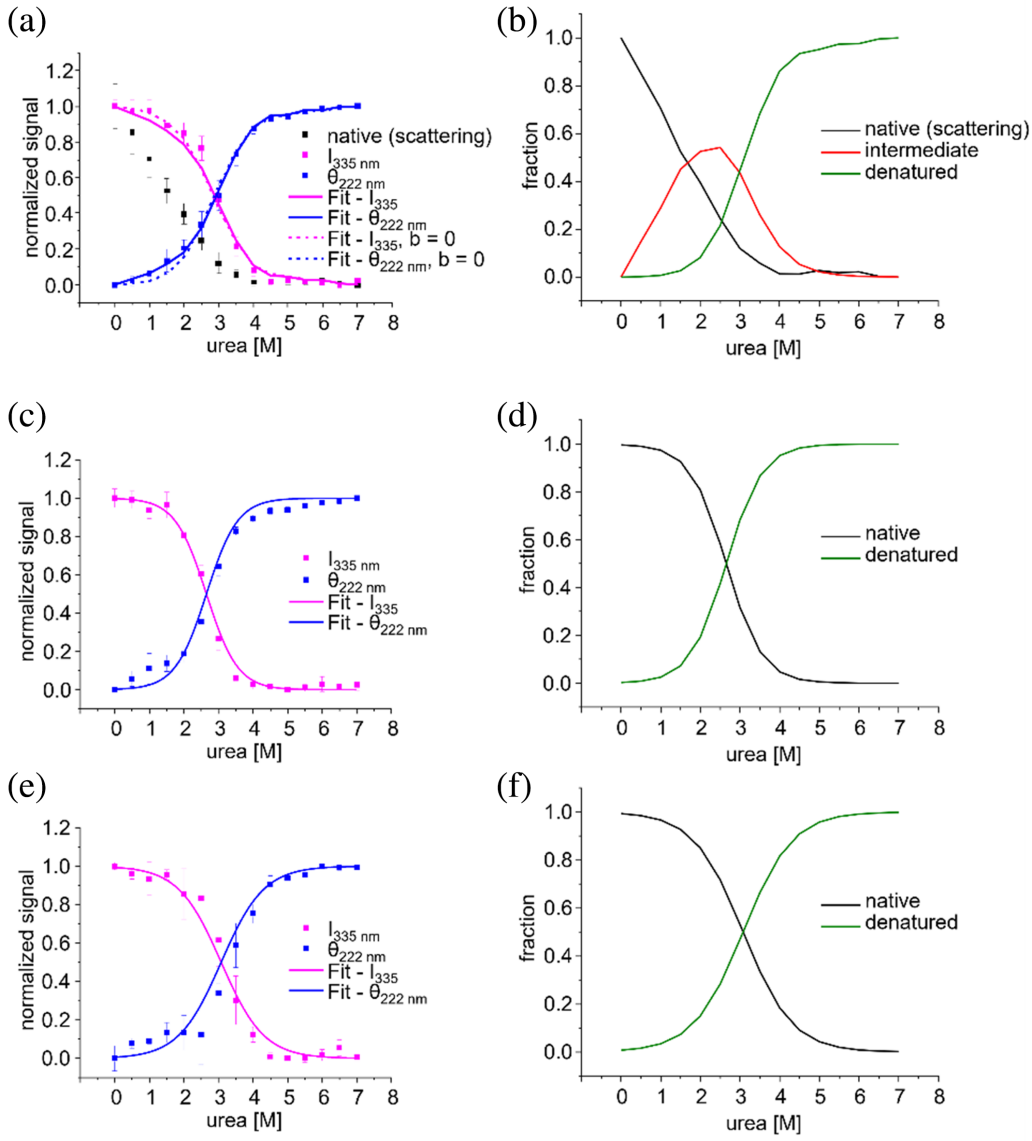
Global fit of experimental data to a 3‐state model of urea‐induced IM30 destabilization. (A) Global fit of a 3‐state model for IM30 wt (a and b) and a 2‐state model for IM30* (c and d) and IM30_26–156_ (e and f). (a, c, and e) normalized Trp intensity and CD signal at 222 nm with normalized experimental data (squares) and corresponding fits (line). (b, d, and f) Calculated fractions of IM30 states at increasing urea concentrations based on the fitting results. Oligomeric barrel structure—native state, equivalent to the normalized scattering signal (black), monomer—intermediate state (red), denatured state (green). The error bars in (a, c, and e) represent SD, *n* = 3.

Fitting our model to the normalized Trp fluorescence and CD signals simultaneously yielded a *c*_50_ of 3.0 ± 0.1 M for IM30 wt (Figure 10a, see Table S1 for a summary of fit parameters). The fraction of IM30 in the intermediate state (I) is maximal at 2.5M urea amounting to about 60% (Figure 10b). Thus, when using urea to destabilize the IM30 structure it is not possible to exclusively disassemble IM30 barrels completely without simultaneously starting to unfold some of the intermediate I further into U, at least under equilibrium conditions.

For modeling the process of IM30* and IM30_26–156_ denaturation, we tentatively applied a simplified two state model between a native (N′) and an unfolded state (U). The native state (N′) of IM30* differs from the native state (N) of IM30 with respect to the oligomeric state at 0M urea, as solely IM30 is present in barrel structures in its native state (N), whereas the native state of IM30* (N′) represents monomers/small oligomers (Heidrich et al., 2016; Junglas et al., 2020). Furthermore, the C‐terminus of IM30* is unstructured (Junglas et al., 2020). We also refrained from setting the native state of IM30* (N′) equal to the IM30 wt intermediate state (I), as we cannot exclude that the structure of N′ differs to some extent from (I) (Junglas et al., 2020).

Thus, any potential contribution of dissociation of small oligomers to the observed denaturation curve was not taken into account in the model. Yet, considering the complete dissociation of the large barrel at 3M urea, it appeared likely that tetramers or smaller sized oligomers are dissociated well below this urea concentration. Indeed, this simplifying model describes the measured curves very well in both cases.

Overall, the denaturation curves of IM30* and IM30_26–156_ are quite similar. However, the analyses revealed slight difference in the stability towards urea: the transition from (N′) to (U) was observed at *c*_50_ = 2.65 ± 0.03M urea for IM30* and at *c*_50_ = 3.1 ± 0.1M urea for IM30_26–156_ (Figure 10c–f). For the IM30 wt, a value similar to IM30_26–156_ was determined with *c*_50_ = 3.0 ± 0.1M.

Taken together, our model is able to capture the characteristic transitions observed in our experiments (Figure 10). Unwinding of the coiled‐coil, monitored by Trp fluorescence changes, and unfolding of the helices occur simultaneously, with a *c*_50_ of around 3M in all three investigated IM30 variants. A similar unfolding behavior of IM30* and IM30_26–156_ was expected, since in both cases the folded entity is mainly the coiled‐coil region. However, the higher molar ellipticity of IM30* compared to the truncated variant indicates that parts outside of amino acids 26–156 contain short and/or temporary α‐helical structures. In case of the wt, the observation that the unfolding process is very similar to unfolding of the truncated variant suggests that the intermediate (I) is a monomer/small oligomer with largely unfolded helices α4‐6. If these regions were still folded after disassembly of the ring, they would have to be similarly stable as the coiled‐coil region in helices α1‐3 to adhere to the observed two‐step unfolding mechanisms. This appears unlikely considering the structure of IM30* with its largely unfolded helices α4‐6^20^ and the completely unfolded structure of the truncated variant α4‐6 (Junglas et al., 2020; Thurotte & Schneider, 2019). Furthermore, the analyses of the experimentally determined curves also indicate that the secondary structures of monomers incorporated in the ring (N) vs. the intermediate (I) differ. In the fitting process not only *c*_50_ was obtained but also two parameters (*a* and *b*) which contain information about the relative value of the specific magnitude of fluorescence (*a*) and CD (*b*) signals for the different states. The fitted parameter *a* = 0.81 ± 0.03 reflects (*F*_I_–*F*_U_)/(*F*_N_–*F*_U_), suggesting that for IM30 wt about 80% of the total fluorescence change measured between 0 and 7M urea occurs upon unfolding of (I), while 20% occurs upon disassembly of the barrel. In this case, based on the analysis of solvent accessibility of Trp71 in the ring and the monomer, indeed some change observed in the steady‐state fluorescence characteristics (Figure 5) can be rationalized. Also, the fluorescence of IM30* in absence of urea seems to be somewhat lower than for IM30 wt (Figure S9). The fitted parameter *b* = 0.24 ± 0.03 reflects (*c*_I_–*c*_N_)/(*c*_U_–*c*_N_), and thus about 24% of the total change in the CD signal measured between 0 and 7M urea occurs upon disassembly of the barrel, while 76% occurs upon unfolding of (I). If the intermediate (I) reflected an isolated monomer that is as structured as in the ring, the parameter *b* would be 0. To test whether a monomer with secondary structure indistinguishable from the wt (when embedded in the ring) is compatible with the data, we modified the fitting routine, and set *b* = 0. The corresponding fitted curves did not capture the changes in secondary structure observed at low urea concentrations (blue dashed curve in Figure 10a). Furthermore, the 24% decrease in the relative CD signal between (N) and (I) nicely agrees with the differences in CD spectra observed at 0M urea for IM30 wt and IM30*: here, IM30 wt has a molar ellipticity about 29% higher than IM30* (Figure 6b), which likely reflects changes between the monomer embedded in the ring (wt) versus the released monomer (IM30*). Overall, these findings strongly support the assumption that the IM30 helices α4‐6 unfold independently and unfolding of helices α4‐6 is not (directly) connected to unfolding of the structured core formed by the helical hairpin α1‐2. Furthermore, the data suggest that helices α4‐6 fold only in the context of barrel formation.

## 3 CONCLUSION

Despite initial studies involving chaperone‐mediated disassembly and destabilizing mutations (Heidrich et al., 2016; Junglas et al., 2020; Liu et al., 2005, 2007; Saur et al., 2017; Thurotte & Schneider, 2019), assembly and disassembly of IM30 oligomeric structures are poorly understood. Yet, to eventually elucidate the IM30 function, these processes need to be delineated. While partial unfolding of IM30 monomers during (or after) barrel disassembly was indicated, based on analysis of IM30 variants, it remained unclear whether α‐helix formation and assembly of oligomeric structures are coupled. Our results now show that the IM30 protein retains the structure of its α‐helical coiled‐coil domain during and after disassembly of the higher‐ordered oligomer. Furthermore, the helices α4‐6 are unstructured when not assembled in a barrel, in excellent agreement with recent analysis of the isolated α4‐6 fragment (Junglas et al., 2020; Thurotte & Schneider, 2019). A clear indication for the occurrence of transiently existing completed folded monomeric species at low concentrations of urea was not found, yet can also not be excluded due to experimental limitations. Thus, even though it might be possible to disassemble oligomeric structures using urea and liberate protomers, our results clearly show that it is impossible to fully destabilize all oligomeric structures without also starting to unfold the coiled‐coil structure in the monomer.

IM30, as well as PspA, are bacterial ESCRT‐III superfamily members that can serve as manageable models to understand assembly of ESCRT‐III proteins in general. Based on the here presented analysis, great care must be taken when trying to select experimental conditions involving destabilized ESCRT‐III‐oligomers, since an equilibrium between oligomeric, intermediate, and unfolded states may need to be accounted for.

Furthermore, the here described analyzes of a bacterial ESCRT‐III protein has important implications for understanding the assembly and dynamic structural (re)arrangements of ESCRT‐III proteins in general. The driving force for ESCRT‐III‐mediated membrane remodeling in eukaryotes has been suggested to involve protomer exchange in hetero‐oligomeric assemblies by Vps4 coupled to ATP‐hydrolysis (Alonso et al., 2016; Carlton & Baum, 2023; McCullough et al., 2018; Mierzwa et al., 2017; Shestakova et al., 2010; Vietri et al., 2020). This was hypothesized to lead to changes in the tendency of membrane bound ESCRT‐III proteins to form flat spirals, buckling spirals and rod‐like structures (Jiang et al., 2022; Pfitzner et al., 2020, 2021). It is still under debate how protomers are recycled after removal from the oligomer by Vps4 and our understanding of these processes is still rather macroscopic and lacks molecular details (Carlton & Baum, 2023; McCullough et al., 2018). Our data strongly suggest that the stable core could function as an anker point for structural rearrangements during membrane remodeling, whereas other regions are likely crucial for the structural flexibility. Likely, for efficient refolding of oligomers, some residual structure that is stable also outside the oligomeric structure, needs to be preserved in the protomer that can be recognized, leading to binding and incorporation into an oligomer. Our results now show that such a stable intermediate structure exists (at least) for the ESCRT‐III family member IM30 after destabilizing the oligomeric structure by urea and that it is constituted by a conserved feature of the ESCRT‐III protein family, the helices α1‐3 coiled‐coil. The unstructured part of the monomer, on the other hand, leads to an entropic penalty in the process of barrel formation. This is expected to lower the thermodynamic stability of the barrel compared to formation from monomers with preformed helices. Since dynamic oligomer (dis)assembly appears to be crucial for the function of ESCRT‐III proteins, the barrel should be not too stable, in agreement with the now observed barrel disassembly at low urea concentration. Thus, the here noted formation of a stable, structured core (helices α1‐3) in combination with highly destabilized parts (helices α0, α4‐6) likely ensures proper and dynamic (dis)assembly of ESCRT‐III oligomers in vitro, and likely also in vivo.

## 4 MATERIALS AND METHODS

### 4.1 Cloning, expression, and purification of IM30

The construction of the plasmid containing the gene coding for IM30 of *Synechocystis* sp. PCC 6803 (pRSET IM30 wt) was described previously (Fuhrmann, Bultema, et al., 2009b). The construction of the plasmid for expression of IM30*, containing the mutations E83A, E84A, F168A, E169A, R170A, and M171A, was described earlier (Heidrich et al., 2016; Junglas et al., 2020). The plasmid for expression of the truncated IM30 variant IM30_26–156_ is based on pRSET IM30 wt and was constructed using QuickChange PCR to introduce a stop codon at the amino acid position 157 and Gibbson cloning to remove the sequence coding for the first helix of *Syn*IM30, finally resulting in pRSET IM30_26–156_.

All protein variants were expressed in *E. coli* BL21 (DE3) grown overnight in LB medium at 37°C. Cells were harvested by centrifugation (3000 × *g*, 4°C), resuspended in 20 mM imidazole purification buffer (300 mM NaCl, 20 mM imidazole, 50 mM phosphate, pH 7.6) and lysed via sonification at 4°C. Cell debris was removed by centrifugation (12,000 × *g*, 4°C) and His‐tagged protein was bound to Ni‐NTA columns and washed with increasing amounts of imidazole (20, 50, 100 mM). In the end, protein was eluted with purification buffer containing 500 mM imidazole. Buffer was exchanged using PD‐10 columns or dialysis, and protein was concentrated using centrifugal filters (Merck, Darmstadt, Germany) with molecular weight cut‐offs of 30 and 10 kDa for IM30 wt and IM30*, respectively. Protein concentrations were determined using the Bradford assay and protein was frozen in liquid nitrogen and stored at −20°C until use.

### 4.2 Static light scattering

Light scattering was used to follow changes in IM30 particle size at urea concentrations ranging from 0 to 7M urea. A solution containing 3.2 μM protein was incubated for 15 min at 20°C at the indicated urea concentrations in 10 mM HEPES buffer, pH 7.6. Scattering was measured at 20°C using a Fluoromax (Horiba Scientific, Kyoto, Japan) instrument with excitation wavelength set to 600 nm, slidt width 2 nm, and emission spectra were recorded from 200 to 700 nm in 1 nm steps, slid width 2 nm. Light scattering was evaluated as the 2nd order Rayleigh scattering at 300 nm by measuring the average of the signal at 300 nm ± 5 nm for each urea concentration.

### 4.3 Trp fluorescence measurements

Trp fluorescence was monitored to follow changes in Trp71 fluorescence at urea concentrations ranging from 0 to 7 M. For each data point 3.2 μM of protein was incubated for 15 min at 20°C in 10 mM HEPES buffer, pH 7.6 at the indicated urea concentration. Trp fluorescence was measured at 20°C using a Fluoromax (Horiba Scientific, Kyoto, Japan) instrument with excitation wavelength set to 280 nm, slidt width 1 nm, and emission spectra were recorded from 300 to 450 nm in 1 nm steps (slid width 3 nm). The resulting spectra were processed by applying a moving average of ±5 nm. The fluorescence intensity at 335 nm, as well as the maximums wavelength of the spectrum were determined at each urea concentration. The denaturation curve based the Trp emission at 335 nm was normalized and both the maximums wavelength and normalized intensity at 335 nm from at least 3 independent measurements were averaged. Measurements of Trp fluorescence were performed directly after measuring the scattering signal with the same sample.

### 4.4 Circular dichroism spectroscopy

Circular dichroism (CD)‐spectroscopy was used to follow changes in the IM30 secondary structure at urea concentrations ranging from 0 to 7M. For each data point, 3.2 μM of protein (0.1 mg/mL for IM30 and IM30*; 0.07 mg/mL for IM30_26–156_) was incubated for 30 min at 20°C at the indicated urea concentration in 10 mM HEPES buffer, pH 7.6. A CD‐spectrum was measured at 20°C using a J‐1500 CD‐spectrometer (JASCO Corporation, Tokyo, Japan) collecting spectra from 190 to 250 nm at a scanning speed of 100 nm/min in 1 nm steps, slid width 1 nm. The resulting spectra were processed by applying a moving average of ±5 nm. The denaturation curves based on the ellipticity at 208 nm and 222 nm were normalized for comparison. As a second measure for structural changes, the molar ellipticities at 208 nm and at 222 nm were normalized and the molar ellipticity ratio 222‐to‐208 nm was determined for each urea concentration. The results of at least 3 independent measurements were averaged.

### 4.5 Unfolding kinetics

The kinetics of unfolding in 2, 3, and 4M urea were monitored via following changes in Trp fluorescence and turbidity. To this end, 20 μM IM30 wt or IM30* in 10 mM Hepes/NaOH, pH 7.6 was rapidly mixed with buffer containing 4, 6, or 8M urea in a 1:1 ratio, employing the SX20 instrument (Applied Photophysics, Leatherhead, UK). For all curves, a cut off filter at 320 nm was used on the emission side. For monitoring the change in Trp fluorescence, the excitation wavelength was set to 280 nm; for monitoring turbidity, it was set to 325 nm. For each condition, the same solution was used for measuring kinetics based on Trp fluorescence and turbidity, respectively. At least three kinetic curves were averaged for each condition. Since at longer time scales photobleaching became visible, for normalization end time points were selected where this contribution remained below about 2%. The starting time point was set to 6 ms, since at shorter times artifacts due to mixing of buffer with urea occurred due to the difference in refractive index.

### 4.6 Modeling urea‐induced IM30 destabilization

We applied a 3‐state model for IM30 oligomer unfolding: the native state, namely the oligomeric barrel (N), an intermediate monomeric state (I) and the final unfolded state (U). For IM30* and IM30_26–156_ a 2‐state model was used, with a native state (N′) and the unfolded state (U).

Our model is based on the linear combination of the signal Y (measured via light scattering, fluorescence, and CD) from specific IM30 state, which can be expressed as:

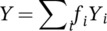

where $Y_{i}$ is the maximum signal that could be produced by species i (i = N, N′, I, U) with *f*_N_ + *f*_I_ + *f*_U_ = 1. For IM30 wt the first step is dissociation of the barrel, which makes a rigorous treatment difficult. However, from the scattering signal we can estimate the overall fraction of non‐oligomers, as demonstrated below, simplifying the model‐based analyses:

Since the small oligomeric/monomeric states and unfolded states are indistinguishable in terms of the scattering signals $\left(S_{I}=S_{U}=S_{I,U}\right)$, we find for the scattering signal of IM30:

(1a) $$S=f_{N}*S_{N}+\left(f_{I}+f_{U}\right)S_{I,U}=S_{I,U}+\left(S_{N}-S_{I,U}\right)f_{N}$$

(1b) $$S_{\mathrm{norm}}=\frac{S-S_{I,U}}{S_{N}-S_{I,U}}=f_{N}$$

Thus, based on the normalized scattering data as a descriptor for the fraction of IM30 in the barrel state, we can replace $f_{N}$ by $S_{\mathrm{norm}}$, and only have to describe the denaturation process by an appropriate equilibrium constant.

The ratio between intermediate and unfolded protein can be expressed in terms of an equilibrium constant, *K*, as:

(2) $$K=\frac{c_{U}}{c_{I}}=e^{\frac{-∆G^{{}^{\circ}}}{\mathrm{RT}}}=e^{-\;\frac{∆G_{H2O}^{{}^{\circ}}-m\left[U\right]}{\mathrm{RT}}}=e^{-\;\frac{C_{50}-\left[U\right]}{d}}$$

We used a similar method as previously described for the determination of $∆G_{H2O}^{{}^{\circ}}$ (Greene & Pace, 1974) by applying the linear extrapolation model (LEM) to parametrize the effect of urea on the denaturation equilibrium constant *K*. Here, *C*_50_ = $∆G_{H2O}^{{}^{\circ}}/m$, defining the urea concentration where *K* = 1, and *d* = *RT*/*m*.

The fractions of smaller oligomeric/monomeric and unfolded states can then be expressed as:

(3a) $$f_{I}=\frac{c_{I}}{c_{N}+c_{I}+c_{U}}=\frac{1}{1+K}\left(1-f_{N}\right)$$

and

(3b) $$f_{U}=Kf_{I}=\frac{K}{1+K}\left(1-f_{N}\right)$$

Here, *c*_N_, *c*_I_, and *c*_U_ are the concentrations of the N, the I, and the U state, and *f*_N_, *f*_I_, *f*_U_ the corresponding fractions of the total protein concentration.

For further analysis, we rearranged the equations describing the contributions of the different species to the fluorescence signal (F) and CD‐signal (D), starting from:

(4a) $$F=f_{N}F_{N}+f_{I}F_{I}+f_{U}F_{U}$$

(4b) $$D=f_{N}D_{N}+f_{I}D_{I}+f_{U}D_{U}$$

In the next step, $f_{U}$ was eliminated from Equation (4a) making use of $f_{U}=1-f_{N}-f_{I}$, yielding

$$F=F_{U}+f_{N}\left(F_{N}-F_{U}\right)+f_{I}\left(F_{I}-F_{U}\right)$$

By normalizing so that $F_{N}=1$ for $f_{N}=1$ we obtain:

$$F_{\mathrm{norm}}=\frac{F-F_{U}}{F_{N}-F_{U}}=f_{N}+af_{I}$$

where $a=\frac{F_{I}-F_{U}}{F_{N}-F_{U}}$.

Analogously, by eliminating $f_{N}$ from Equation (4b) and normalizing so that $D_{\mathrm{norm}}=1$ for $f_{U}=1$, we get:

$$D_{\mathrm{norm}}=bf_{I}+f_{U}$$

where $b=\frac{D_{I}-D_{N}}{D_{U}-D_{N}}$. The fit constants *a* and *b* express the differences between the specific fluorescence and CD signals, and thus, the following equations were fitted to the normalized signals simultaneously:

$$F_{\mathrm{norm}}=S_{\mathrm{norm}}+a\frac{1}{1+K}\left(1-S_{\mathrm{norm}}\right)$$

$$D_{\mathrm{norm}}=\frac{b+K}{1+K}\left(1-S_{\mathrm{norm}}\right)$$

In the cases of IM30* and IM30_26–156_, we consider only two states, namely the smaller oligomeric/monomeric native state (N′) and the unfolded state (U), and analogously we obtain the simplified equations:

$$F_{\mathrm{norm}}=f_{N^{\prime }}=\frac{c_{N^{\prime }}}{c_{N^{\prime }}+c_{U}}=\frac{1}{1+K}$$

and

$$D_{\mathrm{norm}}=f_{U}=\frac{c_{U}}{c_{N^{\prime }}+c_{U}}=\frac{K}{1+K}$$

The model was implemented in OriginLab 2020 and fitted using the Levenberg Marquard algorithm by setting all parameters as shared global fitting parameters for each respective IM30 variant. All fits converged and fit parameters are summarized in (Table S1).

### 4.7 Coarse‐grained simulations

The initial configuration of IM30 wt monomer and IM30* for the simulations were designed by the package HOOBAS (Girard et al., 2019). The residues in the sequence of IM30 were mapped to the PLUM force field (Bereau & Deserno, 2009). The PLUM represents each amino acid as three or four beads N, Cα, C′, and Cβ for amide group, central carbon, carbonyl group and side chain respectively, with special consideration for Gly and Pro residues. This force field has two distinct advantages: it uses implicit solvent which allows for fast dynamics, plus has an explicit H‐bond potential on the backbone. The H‐bond potential is

$$V_{\mathrm{hb}}\left(r,\theta_{N},\theta_{C}\right)=\lambda \epsilon_{\mathrm{hb}}\left[5{\left(\frac{\sigma_{\mathrm{hb}}}{r}\right)}^{12}-6{\left(\frac{\sigma_{\mathrm{hb}}}{r}\right)}^{10}\right]\left(\cos^{2}\theta_{N}\cos^{2}\theta_{C}\right)$$

over the domain |*θ*_N_|, |*θ*_C_| < 90°, and is otherwise zero; *r* is the distance between the two beads N and C′, *σ*_hb_ is the equilibrium distance, θ_N_ is the angle formed by the atoms HNC', *θ*_C_ corresponds to the angle NC'O, and *ϵ*_hb_ is the potential depth for hydrogen bond. Here, *λ* ≤ 1 is a prefactor used to perform denaturation by urea; in the original PLUM force‐field *λ* = 1. The Hamiltonian replica exchange method (HREM) was used to efficiently sample the system configurations (Sugita & Okamoto, 1999). Every 10^5^ time step, we attempted to exchange the configuration of half of the replicas with their neighbor. The standard metropolis criteria were applied, which resulted in random walk of the configurations in *λ*‐space. Twelve replicas were used, with linear biasing from *λ* = 0.75 to *λ* = 1.0. The entire simulation was carried out on HOOMD‐blue (Anderson et al., 2020). Simulations were run in the canonical ensemble (NVT) by means of Langevin dynamics. The time step was set to δ*t* = 0.01*τ*, where *τ* is the unit of time, corresponding to 10 fs. Each simulation was run for 10 μs in total.

The secondary structure determination is based on the backbone dihedral angles (*φ* and *ψ*) (Best & Hummer, 2009). In our study, a residue is considered in helical form if its *φ* (phi) angles fall between −160° and −20°, and its *ψ* (psi) angles fall between −120° and 50° in plot. A residue is part of an α‐helix if it is in a helical form, and both preceding and succeeding amino acids are in helical form. The helical propensity of a residue is then simply defined as the fraction of time it is part of an α‐helix (Zillmer, 2021).

## AUTHOR CONTRIBUTIONS

**Ndjali Quarta:** Conceptualization; data curation; formal analysis; investigation; methodology; software; validation; visualization; writing – original draft; writing – review and editing. **Tika Ram Bhandari:** Investigation; data curation; formal analysis; software; validation; visualization; writing – original draft. **Martin Girard:** Conceptualization; data curation; formal analysis; supervision; funding acquisition; methodology; project administration; resources; software; validation; writing – original draft. **Nadja Hellmann:** Data curation; investigation; software; validation; visualization; writing – original draft. **Dirk Schneider:** Conceptualization; formal analysis; methodology; funding acquisition; supervision; project administration; resources; writing – original draft; writing – review and editing.

## FUNDING INFORMATION

This work was funded by the Max‐Planck Graduate Center at the Max Planck institutes and the University of Mainz, as well as by the Deutsche Forschungsgemeinschaft (DFG, SCHN 690/16‐1 and CRC1551 [project no. 464588647]).

## CONFLICT OF INTEREST STATEMENT

The authors declare no conflict of interests.

## Supporting information


**FIGURE S1:** Model of an IM30 barrel with the third layer of monomers highlighted.
**FIGURE S2:** Model of an IM30 barrel with a single stack of monomers highlighted.
**FIGURE S3:** Influence of relative H‐bond strength on the α‐helicity of IM30* monomers determined via coarse‐grained simulations in solution.
**FIGURE S4:** Trp fluorescence intensity and CD signal at 222 nm at increasing urea concentrations.
**FIGURE S5:** Influence of relative H‐bond strength on the α‐helical propensity of IM30* determined via coarse‐grained simulations of monomers in solution.
**FIGURE S6:** IM30 wt denaturation kinetics.
**FIGURE S7:** Kinetics of IM30* denaturation.
**FIGURE S8:** Normalized IM30 wt denaturation kinetics.
**FIGURE S9:** Trp fluorescence spectra of IM30 and IM30*.
**TABLE S1:** *R*‐values and parameters of global fits used in this study.
