# Supplementary material for: Monomer unfolding of a bacterial ESCRT‐III superfamily member is coupled to oligomer disassembly
Source: Protein Sci. 2024 Oct 29;33(11):e5187. doi: 10.1002/pro.5187 (PMC11520248; doi:10.1002/pro.5187)
Supplement: Supplementary file 1 — FIGURE S1: Model of an IM30 barrel with the third layer of monomers highlighted. FIGURE S2: Model of an IM30 barrel with a single stack of monomers highlighted. FIGURE S3: Influence of relative H‐bond strength on the α‐helicity of IM30* monomers determined via coarse‐grained simulations in solution. FIGURE S4: Trp fluorescence intensity and CD signal at 222 nm at increasing urea concentrations. FIGURE S5: Influence of relative H‐bond strength on the α‐helical propensity of IM30* determined via coarse‐grained simulations of monomers in solution. FIGURE S6: IM30 wt denaturation kinetics. FIGURE S7: Kinetics of IM30* denaturation. FIGURE S8: Normalized IM30 wt denaturation kinetics. FIGURE S9: Trp fluorescence spectra of IM30 and IM30*. TABLE S1: R‐values and parameters of global fits used in this study. [file PRO-33-e5187-s001.pdf]

Supporting Information

Monomer unfolding of a bacterial ESCRT-III superfamily member is coupled to oligomer disassembly

Ndjali Quarta[1], Tika Ram Bhandari[2], Martin Girard[2], Nadja Hellmann[1], Dirk Schneider[1,3]

[1]Department of Chemistry – Biochemistry, Johannes Gutenberg University, Hanns-Dieter-Hüsch-Weg 17, 55128 Mainz, Germany; [2]Max Planck Institute for Polymer Research, Ackermannweg 10, 55128 Mainz, Germany; [3]Institute of Molecular Physiology, Johannes Gutenberg University, 55099 Mainz, Germany

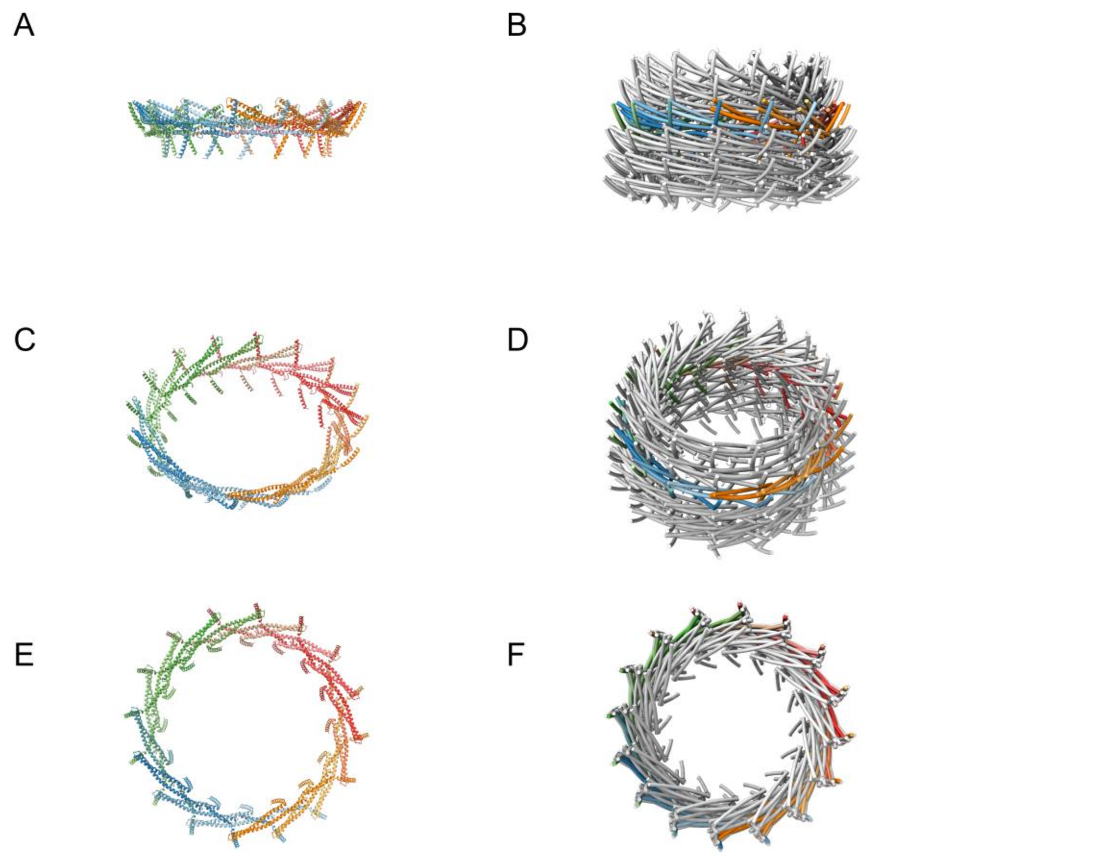

Figure S1: Model of an IM30 barrel with the third layer of monomers highlighted.

(A, B) Front view, (C, D) front view tilted by 45° around the vertical axis towards the viewer, and (E, F) top view (tilted 90° compared to (A, B) around the vertical axis) showing the isolated third layer of monomers in the barrel (A, C, E) and the complete barrel with C16 symmetry (B, D, F). Each monomer of the third layer is differently colored. (PDB:7O3Y)

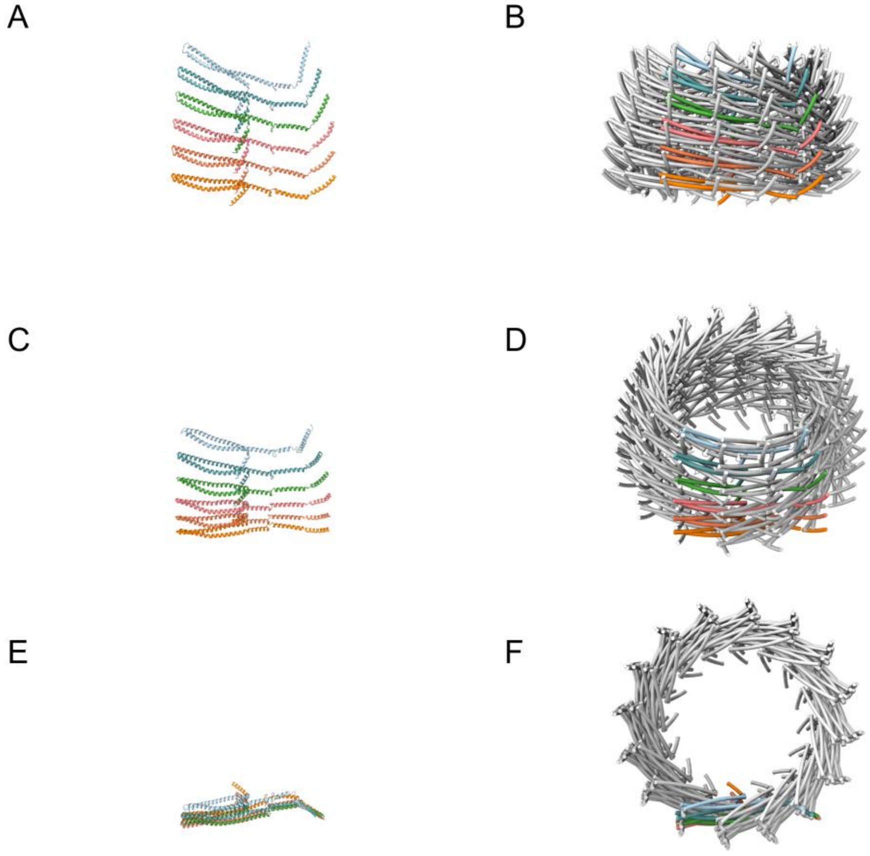

Figure S2: Model of an IM30 barrel with a single stack of monomers highlighted.

(A, B) Front view, (C, D) front view tilted by 45° around the vertical axis towards the viewer, and (E, F) top view (tilted 90° compared to (A, B) around the vertical axis) showing the isolated third layer of monomers in the barrel (A, C, E) and the complete barrel with C16 symmetry (B, D, F). Each monomer of the highlighted stack is differently colored. (PDB:7O3Y)

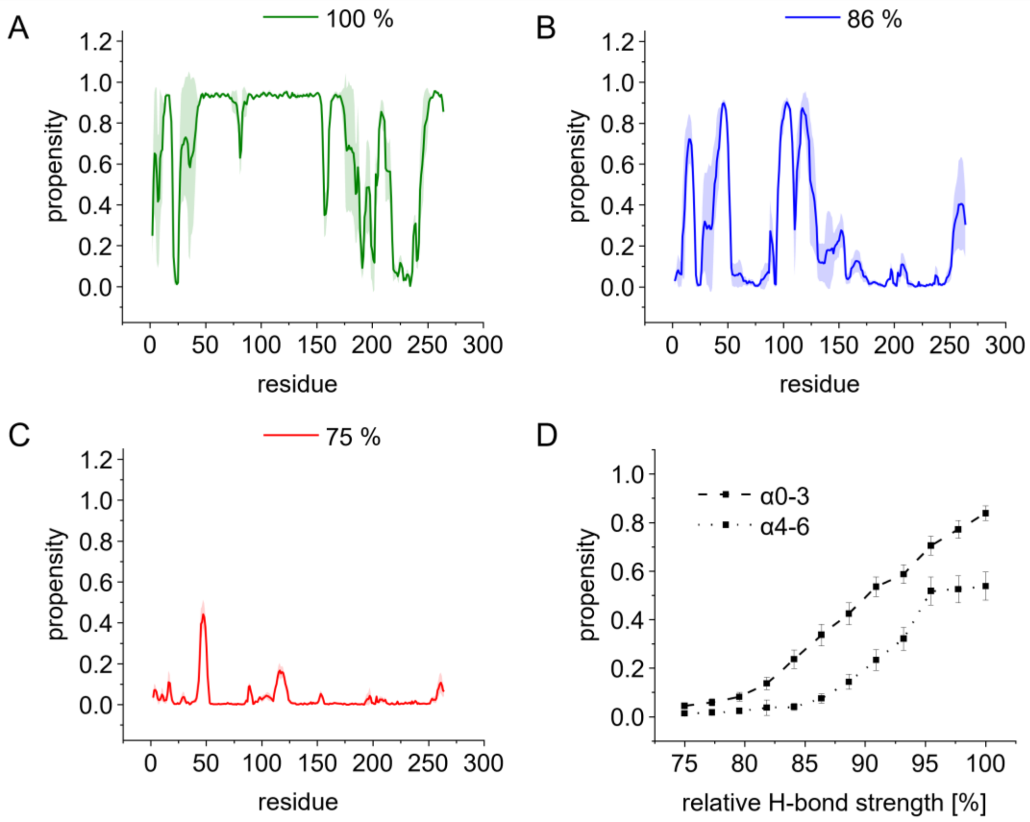

Figure S3: Influence of relative H-bond strength on the α-helicity of IM30* monomers determined via coarse-grained simulations in solution. Sequence propensity *vs*. amino acid position of full-length protein at (A) 100%, (B) 86% and (C) 75% H-bond strength. (D) Helical propensity of fragments α0-3 and α4-6 determined based on simulations of the full length IM30* α0-6.

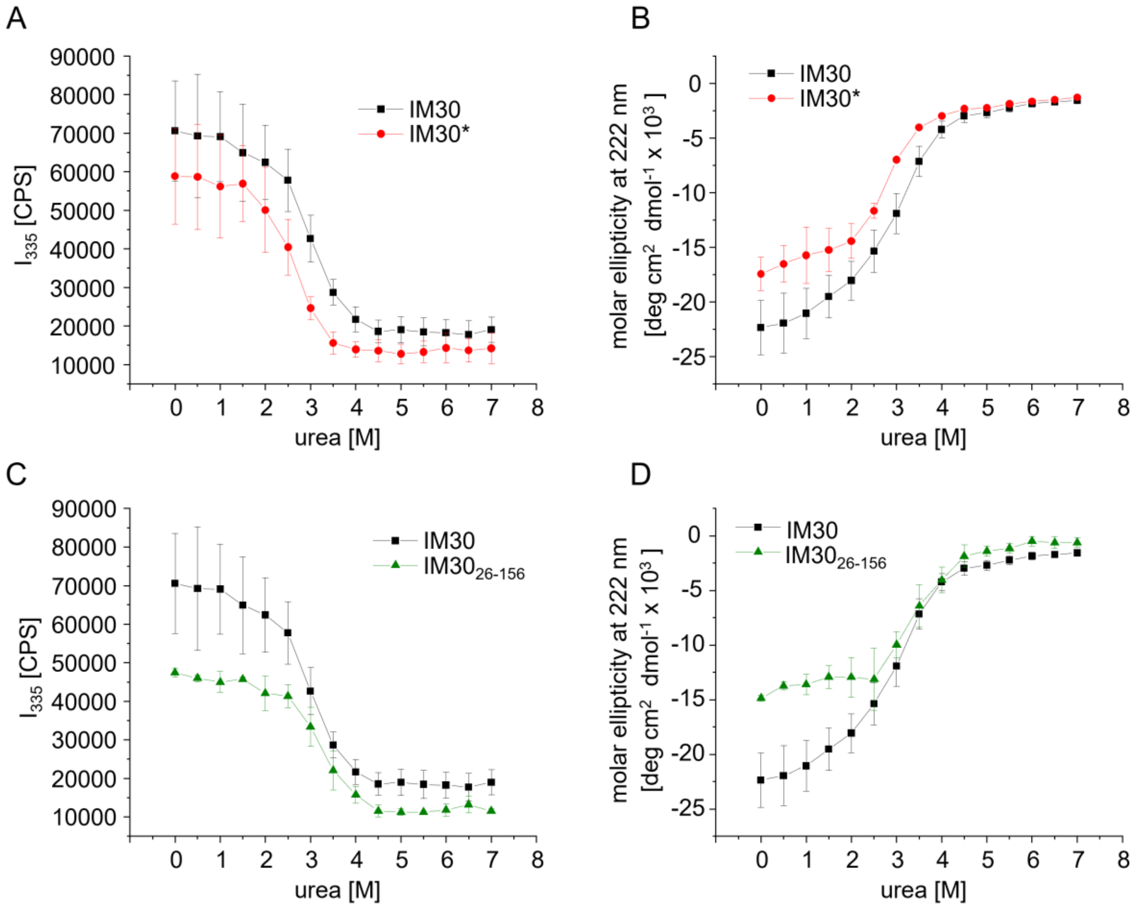

Figure S4: Trp fluorescence intensity and CD signal at 222 nm at increasing urea concentrations.

(A) Trp fluorescence intensity and (B) CD signal at 222 nm of IM30 (black) and IM30* (red). (C) Trp fluorescence intensity and (D) CD signal at 222 nm of IM30 (black) and $IM30_{26\text{-}156}$ (green). The error bars represent SD, n=3.

Note: In case of IM30 the disordered regions contribute only little to the total protein structure whereas the much shorter construct of $IM30_{26\text{-}156}$ has much higher portion of flexible regions (~33%) at 0 M urea compared to IM30. Therefore, the molar ellipticity for the latter seems much lower compared to IM30.

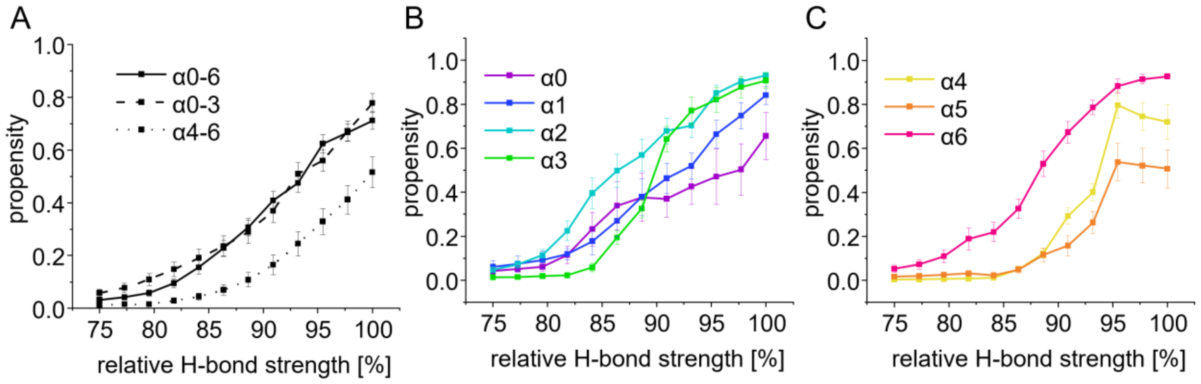

Figure S5: Influence of relative H-bond strength on the α-helical propensity of IM30* determined via coarse-grained simulations of monomers in solution. (A) Propensity of the full length IM30* protein (α0-6) and its fragments (α0-3) and (α4-6) simulated separately. (B, C) Helical propensity of each helix of the α0-3 (C) and α4-6 (D) fragments with respect to the relative H-bond strength calculated from simulating the structure of the full-length IM30* protein α0-6..

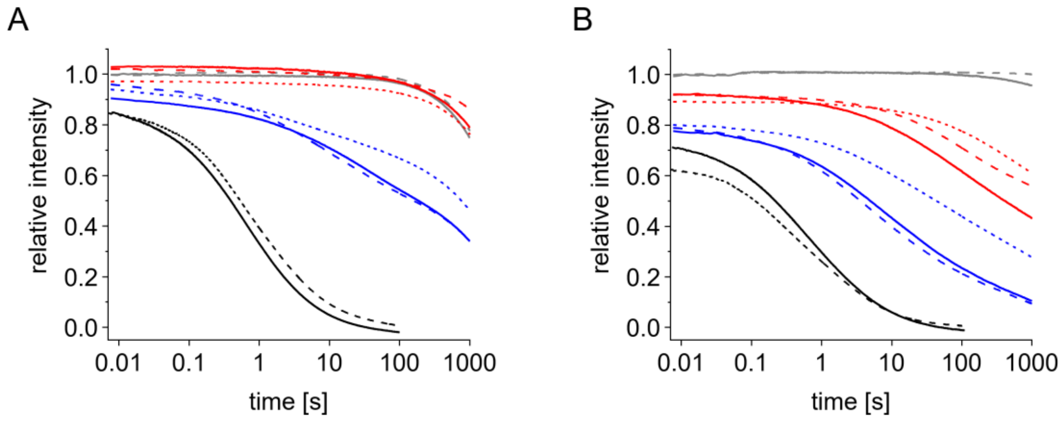

Figure S6: IM30 wt denaturation kinetics.

IM30 wt was mixed 1:1 with a solution containing different urea concentrations, resulting in the following final concentrations: grey: 0 M (control); red: 2 M; blue: 3 M; black: 4 M. The signal of the control at the beginning of the reaction was set as 1, the signal at 100 s measured in presence of 4 M urea was defined as end of the reaction and set as 0. (A) Changes in the Trp-fluorescence; (B) changes in the scattering intensity. The decrease in the Trp-fluorescence intensity observed from around 100 s on in the control is caused by photobleaching. The results of experiments with three different protein preparations are shown. Each individual curve is the mean of 2-3 repetitions.

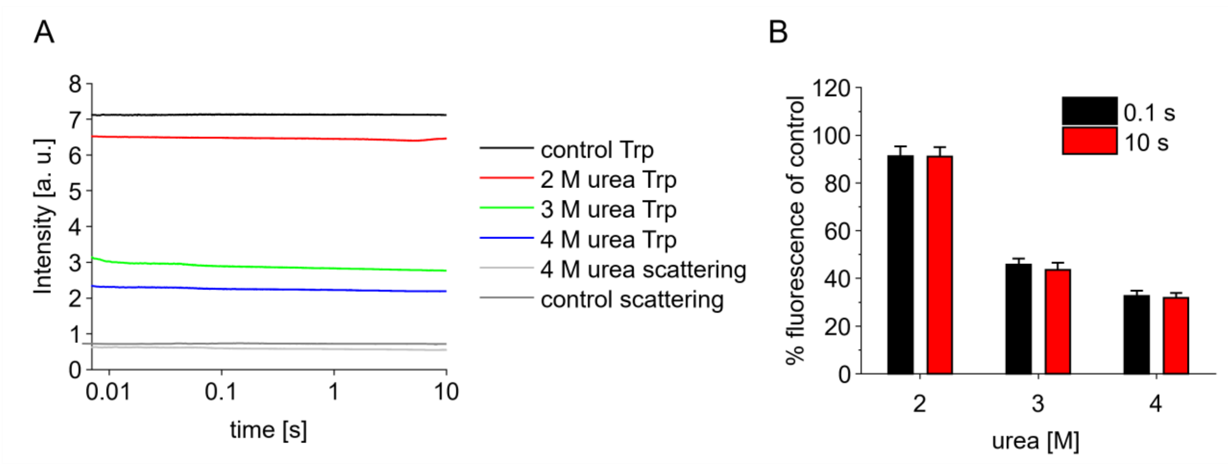

Figure S7: Kinetics of IM30* denaturation.

(A) IM30* was mixed 1:1 with a solution containing different urea concentration, resulting in the following concentrations: black: 0 M (control); red: 2 M; green: 3 M; blue: 4 M and Trp fluorescence changes were monitored. The scattering signal is shown in grey, with no difference for folded (control) or unfolded (4 M) IM30*. The reaction completes within the instrument´s dead time, as indicate by the stable level of the exemplary experimental data shown. This was observed consistently in further measurements, as indicated by the comparison of the fluorescence measured at different urea concentrations, normalized to the corresponding control (B). Here, no difference between values measured at 0.1 s or 10 s was observed at any urea concentration (mean of ≥ 6 individual measurements, presented with the standard deviation).

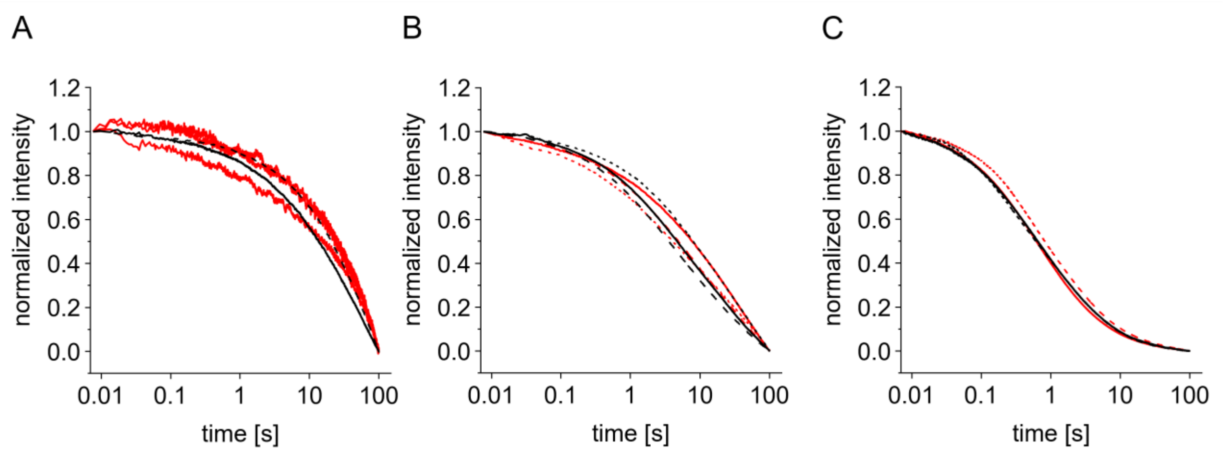

Fig. S8: Normalized IM30 wt denaturation kinetics.

The data shown in Figure 9 in the main text for 2, 3 and 4 M urea are normalized to 1 at the beginning of the reaction (6 ms, deadtime of the instrument) and to 0 at 100 s for (A) 2 M, (B) 3 M, and (C) 4 M urea. The fluorescence signal is shown in red, the scattering signal in black. In all three cases, the overlap is not perfect, yet no systematic behavior (*e.g.* the fluorescence being always above the scattering signal) can be observed. Thus, this comparison does not give clear indications for a transiently populated protein state, the kinetics of oligomer dissociation and denaturation overall follow each other.

Note, that the signal/noise ratio is very poor in case of the Trp fluorescence changes observed in presence of 2 M urea. Thus, it cannot be excluded that some transient state exists here.

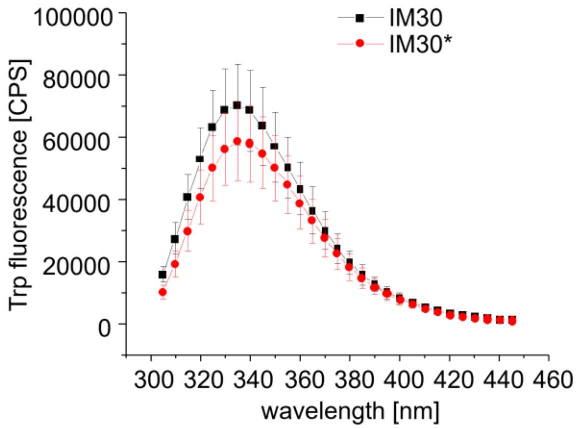

Figure S9: Trp fluorescence spectra of IM30 and IM30*.

Trp fluorescence spectrum of IM30 (black) and IM30* (red) at 0 M urea. The error bars represent SD, n=3.

Table S1: R-values and parameters of global fits used in this study. Parameters *a* and *b* are not applicable for the fits of IM30* and $IM30_{26-156}$.

	IM30wt	IM30wt (b=0)	IM30*	$IM30_{26-156}$
R^2	0.998	0.997	0.998	0.995
a	0.75±0.08	0.93±0.8	-	-
b	0.19±0.6	0	-	-
c_{50}	3.0±0.1	2.7±0.7	2.65±0.03	3.1±0.1
**d**	0.53±0.04	0.62±0.03	0.45±0.02	0.62±0.05
